# The expression profiles and roles of microRNAs in cardiac glucose metabolism

**DOI:** 10.3389/fendo.2025.1565385

**Published:** 2025-07-23

**Authors:** Nan-Nan Shen, Hua Qian, Ya-Fang Zhu

**Affiliations:** ^1^ Department of Pharmacy, Affiliated Hospital of Shaoxing University, Shao Xing, Zhejiang, China; ^2^ Endoscopic center, Affiliated Hospital of Shaoxing University, Shao Xing, Zhejiang, China

**Keywords:** miRNAs, heart, glucose metabolism, systematic review, biomarker

## Abstract

**Background:**

MicroRNAs (miRNAs) are a class of endogenous, non-coding RNAs, that have been implicated in cardiovascular diseases. Recent studies have suggested that dysregulated miRNAs accumulate in the heart and may be associated with impaired cardiac glucose metabolism. However an inconsistent direction of expression was observed in the current available literature. The aim of this study was to characterize miRNA expression profiles associated with glucose metabolism, and to explore their potential as biomarkers for glucose metabolism disorders in diabetic cardiomyopathy (DCM).

**Methods:**

A systematic search of electronic databases, including Embase, PubMed, and the Cochrane Library, was conducted until October 1, 2024. Studies reporting on miRNAs expression profiles that regulate glucose metabolism in the heart were selected for inclusion. Pooled results were presented as log10 odds ratios (logORs) with 95% confidence intervals (CIs), using random-effect models. Subgroup analyses were conducted based on species, region, and sample source. Analyses by species focused specifically on humans and mice. The quality of included articles was assessed using the modified Diagnostic Accuracy Study 2 (QUADAS-2) tool. All workflows, including abstract screening, full-text review, data extraction, and quality assessment, were independently performed by two reviewers.

**Results:**

A total of 47 eligible articles were included in this study, identifying 70 dysregulated miRNAs. Further analysis revealed that compared with the non-DCM group, the DCM group exhibited differential miRNA expression, with 12 miRNAs consistently upregulated and 8 consistently downregulated. Among these miRNAs, miR-199a (logOR 4.59; 95% CI: 3.02-6.15) was the most upregulated and frequently reported (n=7 studies), while let-7 (logOR 4.48; 95% CI: 2.41-6.55) was the most downregulated (4 studies). Subgroup analysis indicated that miRNA-21 was the most upregulated in cardiac tissue, and miRNA-133 was the most downregulated in cardiomyocytes. Additionally, miRNA-21 was found to be the most upregulated across different species. In the region subgroups, miRNA-199a and miRNA-503 were the most upregulated and downregulated in Asian countries, whereas miRNA-378 was the most dysregulated in non-Asian countries.

**Conclusion:**

In summary, this study identified 20 consistently dysregulated miRNAs assocaited with myocardial glucose metabolism. Six dysregulated miRNAs, including miRNA-199a, let-7, miRNA-21, miRNA-133, miRNA-503 and miRNA-378, have potential as candidate miRNA biomarkers of glycometabolism in the heart. These findings require further validation in future larger-scale studies.

## Introduction

The rising prevalence of diabetes mellitus (DM), including type 1, type 2, and other subtypes, poses a significant socioeconomic burden worldwide. Notably, type 2 diabetes mellitus (T2DM) is a metabolic disorder characterized by systemic and myocardial insulin resistance, thereby increasing the risk of cardiovascular complications (1). Diabetic cardiomyopathy, a common complication of diabetes, is marked by myocardial hypertrophy, myocardial fibrosis, and cardiac dysfunction (2–4). While the molecular mechanisms underlying insulin resistance have been extensively studied, the pathophysiology of myocardial insulin resistance remains poorly understood, necessitating further investigation. Abnormal glucose metabolism, observed in patients with diabetic cardiomyopathy, has been identified to be associated with cardiac dysfunction (5). Several key regulators, including GLUT-4, Pyruvate dehydrogenase complex (PDH), Glycogen synthase kinase 3β (GSK-3β), and Insulin-like growth factor 1 receptor (IGF-1R), are critical for cardiac glucose metabolism (6–8).

MicroRNAs (miRNAs), a class of small non-coding RNAs, have been recognized for their role in regulating gene expression through binding to the 3’ untranslated region (3’-UTR) of target mRNAs (9). Previous studies have reported that miRNAs play a crucial role in glucose metabolism across various organs. MiR-146a has been shown to enhance hepatic glucose tolerance by targeting the oxidative metabolism of fatty acids (10). MiR-140-5p mitigates high glucose-induced apoptosis and inflammation in the kidney (11). Furthermore, it has been demonstrated that miRNAs are involved in the development of glucose metabolism in metabolic diseases (12, 13). For instance, miR-29 is dysregulated in muscle, fat, and liver tissues, where it regulates insulin-stimulated glucose uptake (14). Importantly, growing evidence demonstrates that miRNAs play a significant role in the development of various cardiac diseases, particularly in cardiac hypertrophy and glucose metabolism (15, 16). MiR-150 regulates glucose utilization through GLUT-4 in insulin-resistant heart muscle (17). GLUT-4, a key target gene, plays a critical role in myocardial insulin resistance (18).

Taken together, these findings in the current literature highlight the importance of miRNAs in glucose metabolism in the heart. However, the expression profiles of miRNAs across individual studies have yielded inconsistent results. This variability may stem from differences in miRNA sources. For example, miRNA-499 was found to be down-regulated in cardiomyocytes (19), yet up-regulated in myocardium (20). Moreover, even in the same tissue type, the direction of miRNA expression may vary across different studies. Wang et al. indicated a significant downregulation of miR-221 in myocardium (21), whereas another study observed upregulation of miR-221 (20). These conflicting findings underscore the critical impact of heterogeneity among individual studies in miRNA expression profiles. Therefore, this study aims to summarize dysregulated miRNAs in cardiac glucose metabolism, explores their pathological contributions to metabolic dysregulation, and identifies potential biomarkers for myocardial glucose metabolism monitoring.

## Methods

### Search strategy

A comprehensible search was performed across the PubMed, Embase, and Cochrane Library databases to identify relevant miRNA expression profiling articles from inception until October 1, 2024. The following items were used in the title/abstract: (microRNA or miR- or miRNA), (glucose metabolism or glycometabolism), (expression or profiling or profile). Detailed search queries are provided in **Supplementary Table 1**. Furthermore, a manual search was supplemented by screening the reference lists of retrieved studies. Two reviewers independently performed the literature search, and any discrepancies were resolved through discussion with a third reviewer to reach a consensus.

### Literature selection

The retrieved articles were screened to identify eligible studies. After removing duplicates, an initial screening was performed to identify potentially eligible studies according to their titles and abstracts. Afterwards, two investigators independently reviewed the full-text studies based on pre-defined criteria, and any discrepancies were resolved through consensus by a third researcher.

### Inclusion and exclusion criteria

The eligibility criteria were as follows: (1) observational studies (including cohort,

cross-sectional, and case-control studies) that investigated miRNA expression patterns or the diagnostic value of miRNAs in myocardial glucose metabolism; (2) studies must report sample sizes for differentially expressed miRNAs between the normal and abnormal glycometabolism groups; (3) miRNA expression profiles were assessed using techniques such as qPCR, real-time PCR, and microarray; (4) only papers written in English were included. The exclusion criteria were as follows: (1) studies that examined glucose metabolism in organs other than heart (e.g., liver, kidney, adipose tissue, skeletal muscle, etc.); (2) various types of literature, including conference abstracts, case reports, meta-analyses, letters, comments, editorials, and reviews; (3) articles lacking essential data. In cases of duplicate studies from the same research, the study most closely aligned with the inclusion criteria was selected.

### Data extraction and collection

Two reviewers independently extracted essential information from the included articles, with any discrepancies resolved through consensus after in-depth discussion involving a third researcher. The extracted data included the following details: first author, year of publication, country, ethnicity, species, detection methods, sample types, sample size, expressed direction, number of dysregulated miRNAs, and the regulatory mechanism of miRNAs.

### Quality assessment

The Quality Assessment of Diagnostic Accuracy Studies-2 (QUADAS-2) was utilized to assess quality of the included studies. This tool consists of eight questions, each of which is rated as “yes”, “no” and “unclear”. Two independent authors appraised the quality of all eligible articles, and any discrepancies were resolved through discussion with a third reviewer to reach a final consensus.

### Data synthesis and statistical analysis

The extracted data were subjected to statistical analysis using Stata software version 13 (Statacorp, College Station, Texas, United States). Results were presented as log odds ratios (logORs) with corresponding 95% confidence interval (CI), refecting number and direction of dysregulation between the normal and abnormal glucose metabolism groups. Compared to the normal group, logOR values greater than 1 in the abnormal group indicate upregulation. Conversely, a logOR value greater than 1 in the normal group relative to the abnormal group indicates downregulation. A P-value<0.05 was considered statistically significant. Heterogeneity was assessed using the Q test and I^2^ statistics by random-effects model, I^2^< 50% suggests minimal heterogeneity among studies. The significance of dysregulated miRNAs in the abnormal group was ranked based on: (1) number of consistent sub-studies; (2) total sample size; (3) the magnitude of logOR values. Subgroup analysis were performed according to species, ethnicity and tissue type.

## Results

### Literature retrieval and search results

**Figure 1:**
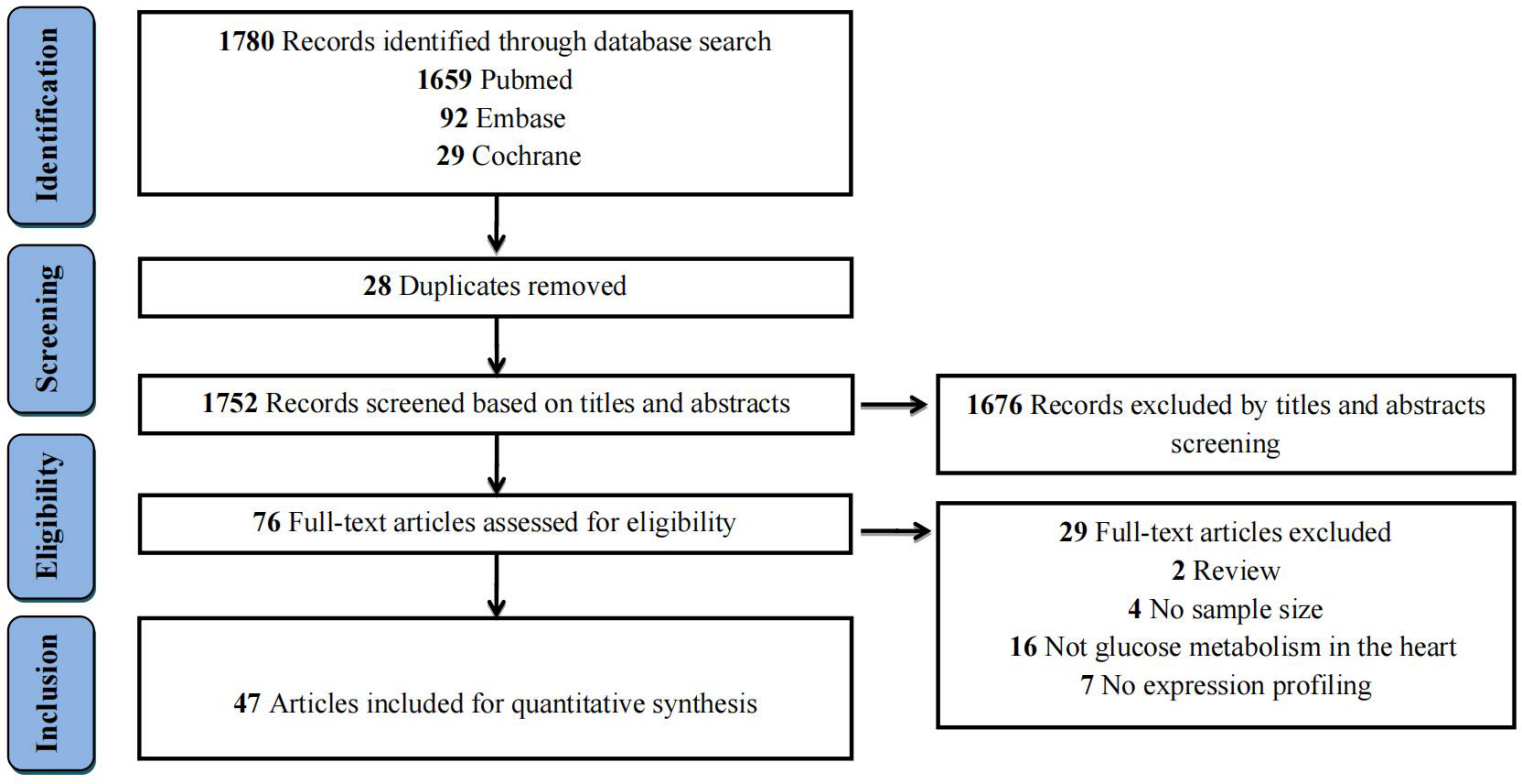
Flow chart of studies selection process for this systematic review.

The literature search process and study selection were illustrated in **Figure 1**. The initial literature search yielded a total of 1,780 records (PubMed = 1,659, Embase = 92, and Cochrane = 29) according to the eligibility criteria (**Supplementary Table 1**). After removing 28 duplicates, the remaining 1,752 records were further screened based on their titles and abstracts. Subsequently, 76 full-text articles were assessed for eligibility. Ultimately, 47 studies were selected for the quantitative analysis. The specific reasons for excluding studies were displayed in **Supplementary Table 2**.

### Study characteristics

All the included studies were published between 2009 and 2023, and quantitative real-time PCR (qRT-PCR) was used as the technique for evaluating miRNA expression in all studies. The number of differentially expressed miRNAs in individual studies ranged from 1 to 16, with the majority of miRNAs showing upregulation in studies of abnormal glucose metabolism. Sample sizes varied from 6 to 120 across the studies. Different specimen types were utilized, primarily including cardiomyocyte and myocardium. Detailed characteristics of the 47 eligible studies are provided in **Table 1**.

**Table 1:** Main characteristics of miRNA expression from the included studies.

First Author	Country	Tissue	Method	Differentially expressed microRNAs			
				Sample size Case/Control	Total	Increased	Decreased
Zuo et al., 2016 (22)	China	cardiomyocyte	qRT-PCR	3/3	1	1	0
Zhu et al., 2017 (23)	China	cardiomyocyte	qRT-PCR	6/6	1	0	1
Zhang et al., 2017 (24)	China	cardiomyocyte	qRT-PCR	6/6	1	1	0
Zhang et al., 2013 (25)	China	cardiomyocyte/myocardium	qRT-PCR	8/8	1	0	1
Zhang et al., 2015 (26)	China	cardiomyocyte	qRT-PCR	5/5	1	1	0
Zhang et al., 2018 (7)	China	myocardium	qRT-PCR	11/5	1	1	0
Yu et al., 2021 (27)	China	myocardium	qRT-PCR	5/5	1	1	0
Yang T et al., 2019 (28)	China	cardiomyocyte	qRT-PCR	6/6	1	1	0
Yang Y et al., 2019 (29)	China	myocardium	qRT-PCR	9/9	1	1	0
Yan et al., 2015 (30)	China	myocardium	qRT-PCR	17/13	1	1	0
Xu et al., 2021 (31)	China	myocardium	qRT-PCR	3/3	2	1	1
Wu J et al., 2019 (32)	China	cardiomyocyte/myocardium	qRT-PCR	12/12	1	1	0
Wu N et al., 2019 (33)	China	cardiomyocyte	qRT-PCR	6/6	1	0	1
Wu et al., 2023 (34)	China	myocardium	qRT-PCR	10/10	1	0	1
Wei et al., 2014 (35)	China	myocardium	qRT-PCR	8/8	1	1	0
Wang et al., 2009 (6)	China	cardiomyocyte/myocardium	qRT-PCR	33/33	11	9	2
Wang et al., 2023 (36)	China	myocardium	qRT-PCR	30/30	10	5	5
Trotta et al., 2018 (37)	Romania	cardiomyocyte	qRT-PCR	9/9	1	0	1
Ruiz-Velasco et al., 2020 (38)	UK	myocardium	qRT-PCR	8/8	1	1	0
Park et al., 2018 (39)	Korea	cardiomyocyte	qRT-PCR	6/6	1	1	0
Nagalingam et al., 2013 (40)	USA	myocardium	qRT-PCR	6/6	1	0	1
Mallat et al., 2014 (41)	France	cardiomyocyte	qRT-PCR	3/3	1	0	1
Lu et al., 2010 (42)	UK	myocardium	qRT-PCR	6/6	3	3	0
Lu et al., 2020 (43)	China	myocardium	qRT-PCR	12/12	1	1	0
Long et al., 2013 (44)	China	cardiomyocyte	qRT-PCR	4/4	1	0	1
Liu et al., 2019 (45)	China	cardiomyocyte	qRT-PCR	22/22	1	1	0
Liu et al., 2020 (46)	China	Pericardial fluid	qRT-PCR	60/60	3	0	3
Li et al., 2017 (47)	China	myocardium	qRT-PCR	3/3	1	1	0
Li et al., 2020 (10)	China	cardiomyocyte	qRT-PCR	3/3	2	1	1
Li et al., 2016 (48)	China	myocardium	qRT-PCR	7/6	1	0	1
Lei et al., 2020 (49)	China	cardiomyocyte	qRT-PCR	3/3	1	1	0
Kim et al., 2013 (50)	USA	myocardium	qRT-PCR	5/5	1	0	1
Ju et al., 2020 (17)	China	cardiomyocyte/myocardium	qRT-PCR	12/12	1	1	0
Horie et al., 2009 (51)	Japan	cardiomyocyte	qRT-PCR	6/6	1	0	1
He et al., 2014 (52)	China	cardiomyocyte	qRT-PCR	5/5	1	1	0
Guedes et al., 2016 (19)	Brazil	cardiomyocyte	qRT-PCR	5/5	9	5	4
Gong et al., 2019 (53)	China	myocardium	qRT-PCR	5/5	1	0	1
Fan et al., 2020 (54)	China	cardiomyocyte	qRT-PCR	5/5	1	0	1
Du et al., 2015 (55)	China	cardiomyocyte	qRT-PCR	5/5	1	0	1
Dong et al., 2019 (56)	China	cardiomyocyte	qRT-PCR	3/3	2	0	2
Dong et al., 2018 (57)	China	cardiomyocyte	qRT-PCR	12/12	1	1	0
Diao et al., 2011 (20)	China	myocardium	qRT-PCR	3/3	16	11	5
Das et al., 2012 (58)	USA	cardiomyocyte	qRT-PCR	5/5	1	0	1
Borden et al., 2019 (59)	USA	myocardium	qRT-PCR	3/3	1	1	0
Baseler et al., 2012 (60)	USA	myocardium	qRT-PCR	4/4	4	4	0
Bartman et al., 2017 (61)	USA	myocardium	qRT-PCR	3/3	1	1	0
Arnold et al., 2014 (62)	USA	cardiomyocyte	qRT-PCR	4/4	3	3	0

UK, United Kingdom; USA, United States of America; RT-PCR: reverse transcription- polymerase chain reaction.

### Quality assessment results

The quality of all included literature was assessed by The QUADAS-2 tool. Detailed information and results of quality assessment was presented in **Supplementary Table 3**. The validated and enhanced methodological standards were applicable to the eligible studies. The evaluation results indicated that the overall risk of bias was low, and the included studies met the majority of quality appraisal criteria.

### Results of the dysregulated miRNAs in overall analysis

**Figure 2:**
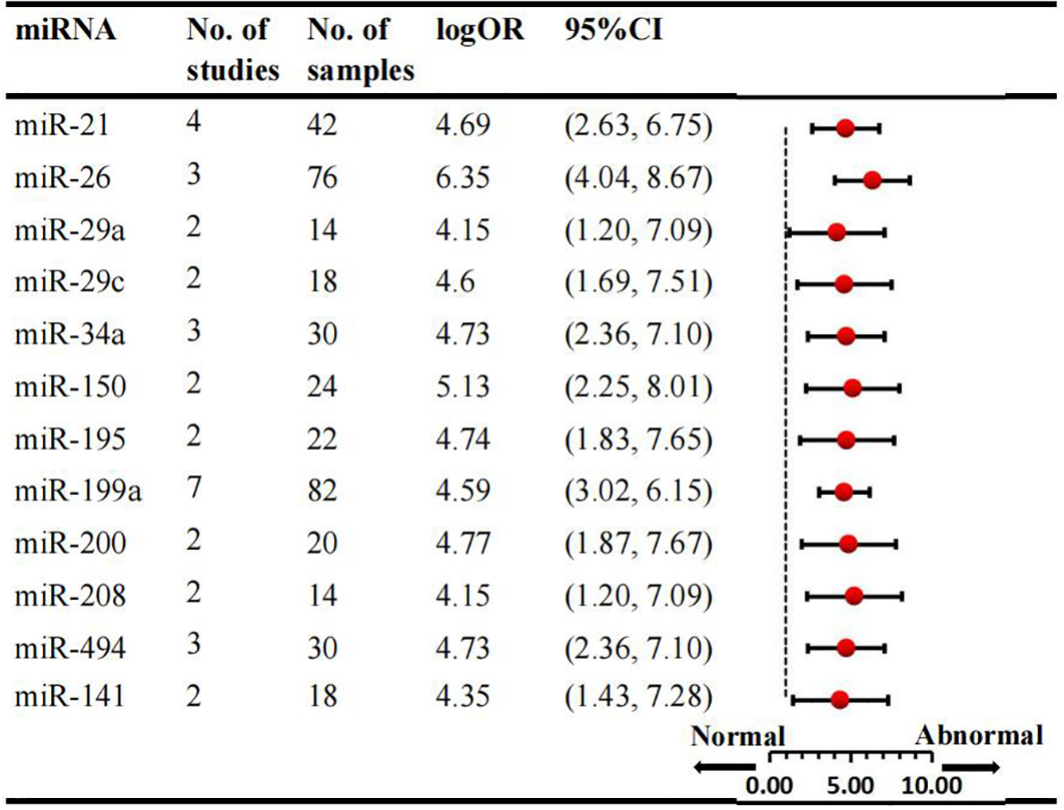
Consistently upregulated miRNAs in overall analysis. miR: microRNA; No.: number of included studies.

**Figure 3:**
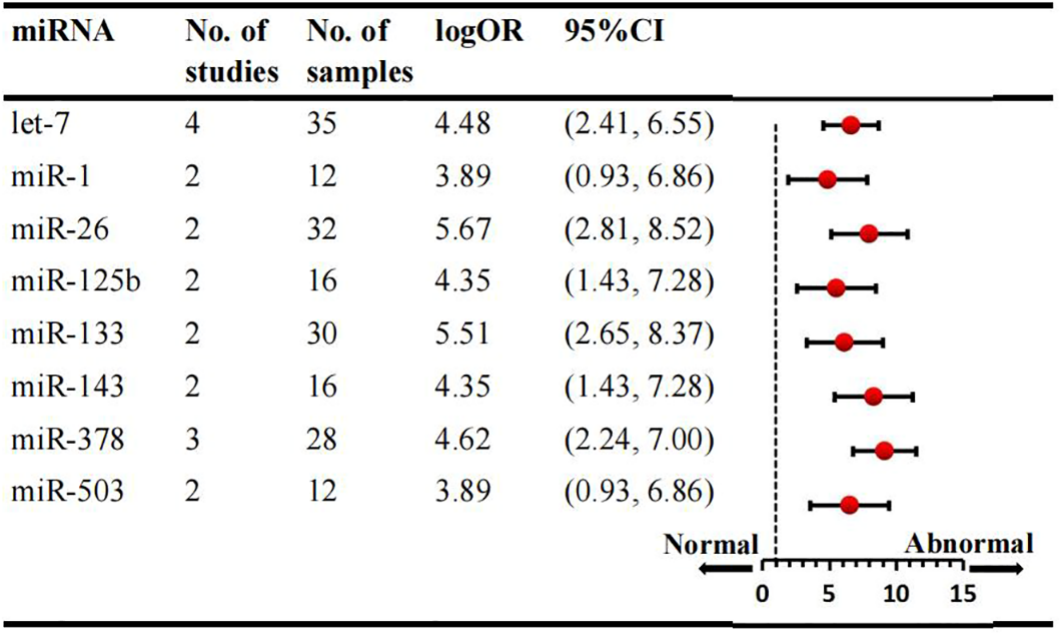
Consistently downregulated miRNAs in overall analysis. miR: microRNA; No.: number of included studies.

We conducted a comprehensive analysis of 47 articles encompassing 70 dysregulated miRNAs comparing the normal glycometabolism group with the abnormal glycometabolism group. Among these miRNAs, 20 (12 upregulated and 8 downregulated) were reported in two or more studies (**Figures 2**, **3**). Detailed information for each miRNA is provided in **Supplementary Figures 1-20**. Additionally, 50 dysregulated miRNAs (34 upregulated and 16 downregulated) were reported only once (**Supplementary Table 4**). Based on the results from 7 sub-studies involving 82 samples, miRNA-199a (logOR 4.59; 95% CI: 3.02-6.15) was identified as the most significantly upregulated miRNA, followed by miRNA-21 (logOR 4.69; 95% CI: 2.63-6.75) due to myocardial glucose metabolism disorder. The most frequently reported downregulated miRNAs were let-7 (logOR 4.48; 95% CI: 2.41-6.55), followed by miRNA-378 (logOR 4.62; 95% CI: 2.24-7.00).

### Results of subgroup analysis

Subgroup analysis was conducted according to sample source, which included tissue and cell samples. Six studies examined miRNAs in myocardial tissue, while four studies focused on miRNAs in myocardial cells. Overall, three consistently upregulated miRNAs (miRNA-21, miRNA-195, miRNA-208) were identified as aberrantly expressed in myocardial tissue samples, with miRNA-21 being the most upregulated (logOR 4.69; 95% CI: 2.63-6.75). The summary results of the tissue source subgroup were presented in **Supplementary Table 5**. Sub-analyses based on species revealed that the number of studies in mice was the largest. Consequently, three consistantly upregulated miRNAs were identified, and the detailed results are provided in **Supplementary Table 6**. Among the animal studies, several miRNAs were consistently upregulated in two studies, including miRNA-21, miRNA-29a, miRNA-208, with the miRNA-21 being the most upregulated (logOR 4.69; 95% CI: 2.63-6.75). Subgroup analyses by ethnicity were conducted for Asian and non-Asian countries. When examining various species types, 8 dysregulated miRNAs were identified in Asian countries and 3 miRNAs in non-Asian studies. The expression signature of miRNAs is region-specific. In the Asian subgroup, 4 miRNAs were upregulated and 4 were downregulated, while in the non-Asian subgroup, 3 miRNAs were upregulated (**Supplementary Table 7**). Notably, miRNA-199a (logOR 4.59; 95% CI: 3.02-6.15) was consistently increased, and miRNA-26 (logOR 5.67; 95% CI: 2.81-8.52) was downregulated in Asian studies. In non-Asian countries, miRNA-29c was upregulated, and miRNA-378 was significantly downregulated.

### Regulatory mechanisms of miRNAs in cardiac glucose metabolism

Dysregulated miRNAs have been identified to play critical roles by regulating glucose metabolism in the heart. The specific roles of miRNAs in cardiac glucose metabolism were summarized in **Table 2**. For instance, miRNA-200, miRNA-223, miRNA-150, and miRNA-141 regulate glucose transport via modulating myocardial GLUT4 expression. Additionally, multiple miRNAs, including miRNA-26, miRNA-99b-3p, miRNA-335, and miRNA-26a regulate glycogenesis by targeting Glycogen synthase kinase 3β (GSK-3β) in the heart. Furthermore, miRNA-34a has been reported to be involved in glycolysis process in the heart. Notably, miRNA-195 enhances the aerobic oxidation of glucose in the myocardium by increasing the acetylation of pyruvate dehydrogenase (PDH), which promotes the conversion of pyruvate and NAD+ into acetyl-CoA.

**Table 2:** Roles of microRNAs in the glucose metabolism in the heart.

Validated targets	miRNA(s)	Study	Key role	Down/Up
Pyruvate dehydrogenase kinase 1(PDK1)	miR-138	Zhu et al., 2017 (23)	Inhibit glycolysis and promotes mitochondrial respiration	down
Lactate dehydrogenase A (LDHA)	miR-34a	Zhang et al., 2017 (24)	Involved in glycolysis process	up
	miR-378	Kim et al., 2013/Mallat et al., 2014 (41)	Balance between oxidative phosphorylation and glycolysis	down
Glycogen synthase kinase 3β (GSK-3β)	miR-99b-3p	Yu et al., 2021 (27)	Involved in glycogen synthesis	up
	miR-26	Zhang et al., 2013/Lu et al., 2020 (25)/ (43)	Involved in glycogen synthesis	down
	miR-335	Wu N et al., 2019 (33)	Involved in glycogen synthesis	down
	miR-199a	Liu et al., 2019/Li et al., 2017/Zuo et al., 2016 (45)/ (47)/ (22)	Involved in glycogen synthesis	up
	let-7	Guedes et al., 2016 (19)	Involved in glycogen synthesis	down
	miR-26a	Park et al., 2018 (39)	Involved in glycogen synthesis	up
	miR-378	Nagalingam et al., 2013 (40)	Involved in process of myocardial fibrosis	down
	miR-29c	Guedes et al., 2016 (19)	Involved in glycogen synthesis	up
	miR-143	Guedes et al., 2016 (19)	Involved in glycogen synthesis	down
	miR-125b	Fan et al., 2020 (54)	Involved in glycogen synthesis	down
	miR-322	Dong et al., 2019 (56)	Involved in glycogen synthesis	down
	miR-503	Dong et al., 2019 (56)	Involved in glycogen synthesis	down
Pyruvate dehydrogenase complex (PDH)	miR-195	Zhang et al., 2018 (7)	Increase acetylation of PDH and ATP synthase	up
Stress-related selenoproteins	miR-200	Yang T et al., 2019 (28)	Lead to glucose metabolism disorder	up
Histone deacetylase 8 (HDAC8)	miR-21	Yan et al., 2015 (30)	Attenuate cardiac hypertrophy	up
Period circadian clock 2 (PER2)	miR-21	Bartman et al., 2017 (61)	Facilitates glycolysis and cardioprotection	up
Lactate dehydrogenase-A (LDHA)	miR-34a	Xu et al., 2021	Regulate glucose metabolic enzymes	up
Estrogen-related receptorβ (ERRβ)	miR-1	Wei et al., 2014 (35)	Lead to glycogen storage and cardiac dilation	up
Insulin-like growth factor 1 receptor (IGF-1R)	miR-503	Wang et al., 2009 (6)	Regulate insulin sensitivity	down
Insulin receptor substrate (IRS)	let-7	Li et al., 2016 (48)	Regulate glucose metabolism	down
	miR-128-3p	Ruiz-Velasco et al., 2020 (38)	Regulate insulin resistance	up
	miR-494	Wu J et al., 2019	Regulate insulin sensitivity	up
Glucose transporter 1/4 (GLUT1/GLUT4)	miR-223	Lu et al., 2010 (42)	Increase GLUT1/GLUT4 glucose transporters	up
	miR-133	Trotta et al., 2018/Horie et al., 2009 (37)/ (51)	Increase GLUT1/GLUT4 glucose transporters	down
	miR-34a	Lu et al., 2010 (42)	Increase GLUT1/GLUT4 glucose transporters	up
	miR-150	Ju et al., 2020 (17)	Regulate glucose metabolism	up
Solute carrier family 25 member 3(Slc25a3)	miR-141	Baseler et al., 2012 (60)	Regulate glucose metabolism	up
	miR-200	Baseler et al., 2012 (60)	Regulate glucose metabolism	down
Iron-sulfur cluster assembly proteins ISCU1/2	miR-210	He et al., 2014 (52)	Suppress proteins ISCU1/2	up

## Discussion

miRNAs are a class of small non-coding RNAs that modulate gene expression by pairing with the 3’-untranslated region (3’UTR) of target mRNAs. Accumulating evidence suggests that miRNAs play pivotal roles in multiple facets of cardiac diseases, including myocardial injury, cardiac fibrosis, and heart failure (63, 64). Notably, prior studies have demonstrated that dysregulated miRNAs in the heart significantly influence cardiac glucose homeostasis (15, 65). However, a significant challenge remains the inconsistency in miRNA expression profiles across different studies. To date, there is a paucity of research providing a comprehensive overview of dysregulated miRNAs involved in myocardial glucose metabolism regulation. Consequently, we undertook an integrative analysis to summarize the differentially expressed miRNAs implicated in myocardial glucose metabolism regulation, based on the available evidence.

In this study, we identified 20 consistently dysregulated miRNAs involved in cardiac glucose metabolism. Among theses, the expression levels of twelve miRNAs were elevated, whereas those of eight miRNAs was reduced. Further analysis revealed that six miRNAs, miRNA-199a, let-7, miRNA-21, miRNA-133, miRNA-503, and miRNA-378, were recognized as potential biomarkers and deemed crucial in the pathogenesis of myocardial glucose metabolism disorder. MiRNAs exhibit differential expression in the cardiovascular system, and play a regulatory role in the pathophysiology of cardiovascular diseases (66). Under normal physiological conditions, heart requires a continuous energy supply to support electrical and mechanical functions, primarily generated through mitochondrial oxidative phosphorylation (67). The involvement of miRNAs in cardiovascular diseases via the regulation of glucose metabolism has been extensively investigated. Previous studies have elucidated the mechanisms by which miRNAs influence various pathological processes, including glucose transport, glycolysis, aerobic oxidation of glucose, and glycogenesis in the heart (22–25).

It is well established that the enhanced glucose uptake primarily results from the translocation of glucose transporter 4 (GLUT-4) and GLUT-1 from intracellular compartments to the surface of cardiomyocytes (68, 69). Upon insulin stimulation, GLUT-4 translocates from the intracellular vesicles to the sarcolemma, thereby increasing glucose uptake and transport (70). Studies have shown that the expression and translocation of GLUT-4 in cardiac myocytes are regulated by miRNAs. Specifically, miRNA-133 and miRNA-223 modulate glucose uptake in cardiomyocytes by targeting GLUT-4 (42, 51). MiRNAs could affect glucose transport in cardiomyocyte hypertrophy. For instance, miRNA-133 has been found to reduce KLF15 expression, a direct upstream regulator of GLUT-4 (51), and decreased level of miR-133a lead to reduced GLUT-4 glucose transporters on the cell membranes in hypertrophic cells (37). Furthermore, cardiac glucose uptake is diminished due to decreased GLUT-4, contributing to impaired myocardial glucose utilization in diabetic cardiomyopathy (37). Additionally, the upregulation of let-7 family enhances glucose utilization via GLUT-4 pathways (48). In summary, dysredulated miRNAs play a crucial role in the regulation of glucose transport in cardiomyocytes.

Cardiomyocytes primarily produce ATP through the glycolysis of glucose, and myocardial glycolysis has been found to convert glucose to macromolecular precursors (71). Several studies have reported on the role of differentially expressed miRNAs in myocardial glycolysis. Mallet et al. discovered that miRNA-378 regulates cardiac energy metabolism by balancing oxidative phosphorylation and glycolysis (41). MiRNAs also influence glycolysis to regulate cardiac function under conditions of myocardial ischemia (59). Upregulated miRNA-21 facilitates increased glycolysis via Per2-dependent mechanisms in myocardial ischemia (61). It is noteworthy that upregulation of miRNA-195 modulates cardiac energy metabolism by directly targeting the pyruvate dehydrogenase complex (PDH) (7). Oxidative phosphorylation of glucose sustains energy necessary for cardiomyocyte function, and multiple miRNAs have been identified as regulators of mitochondrial function in the heart. For instance, miRNA-30 influences apoptosis by targeting the mitochondrial fission machinery (72), while overexpression of miRNA-761 suppresses mitochondrial fission and reduces cardiomyocyte apoptosis (44).

In addition to glucose consumption, excess glucose can be converted into glycogen to provide the high energy demands of the heart (73, 74). A study by Wei et al. demonstrated that the downregulation of miRNA-1 altered glycolysis and glycogenesis by upregulating the expression of related genes (35). Furthermore, miRNAs can target key enzymes in glycogenesis, such as glycogen synthase kinase-3α (GSK3α) and glycogen synthase kinase-3β (GSK3β) in various cardiac pathological processes involving glycogen synthesis. Several miRNAs, miRNA-21, miRNA-199a, miRNA-26, miRNA-378, and miRNA-29c have been shown to regulate the development of pathological cardiac hypertrophy by targeting GSK3β (19, 25, 30, 40, 47). Additionally, other miRNAs, including miRNA-34a, miRNA-199a, and miRNA-26a, affect myocardial ischemia/reperfusion injury via the GSK3β pathway (33, 43, 45, 75). However, the explicit mechnism by which these miRNAs are involved in glucose metabolism in the heart remain to be elucidated.

According to the current inconsistency in miRNA expression profiles, it is crucial to summarize the role of miRNAs in cardiac glucose metabolism. This study evaluated miRNA expression signatures during the pathological process of myocardial glucose metabolism and identified six miRNAs that may serve as potential biomarkers in glycometabolism. Systematic evaluation serves as a robust framework for addressing complex clinical challenges, generating evidence-based solutions through rigorous synthesis of available data (36, 76–79). While a comprehensive analysis of miRNA levels based on available evidence is a significant strength, several limitations must be acknowledged. First, the limited number of individual studies included in the pooled analysis weakens the robustness of conclusions. To ensure statistical power and reliability, we integrated data from 47 articles. Second, due to the insufficient sample size, studies with small sample sizes were not excluded. Finally, the results presented in this article are preliminary, and there is a scarcity of research explicitly investigating the pathological roles of differentially expressed miRNAs in cardiac glucose metabolism. Further experimental validation is essential to ascertain the role of these miRNAs in glycometabolism. Overall, our findings suggest that these miRNAs have the potential to serve as reliable biomarkers for myocardial glycometabolism. Nevertheless, caution is advised in interpreting these results, and rigorous experimental verification remains essential in future investigations.

## Conclusion

In conclusion, this study identified 20 significantly dysregulated miRNAs. Specifically, miRNA-199a, let-7, miRNA-21, miRNA-133, miRNA-503, and miRNA-378 may serve as potential biomarkers for myocardial glucose metabolism. However, their clinical feasibility and applicability remain to be validated, and further investigations is necessary to elucidate the underlying mechanism of these dysregulated miRNAs in cardiac glucose metabolism.

## Data Availability

The original contributions presented in the study are included in the article/**Supplementary Material**. Further inquiries can be directed to the corresponding authors.
