# Supplementary material for: The expression profiles and roles of microRNAs in cardiac glucose metabolism
Source: Front Endocrinol (Lausanne). 2025 Jul 23;16:1565385. doi: 10.3389/fendo.2025.1565385 (PMC12325018; doi:10.3389/fendo.2025.1565385)
Supplement: Supplementary file 1 [file DataSheet1.docx]

SUPPLEMENTAL FILE

Title: Integrated expression profiles and functional crosstalk of microRNAs in cardiac glucose metabolism.

Contents

**Tables**

[Supplementary Table 1. Search strategy used on](#_Toc531790313) [October 28th, 2024 3](#_Toc531790313)

[Supplementary Table 2. Excluded studies with reasons 4](#_Toc531790314)

[Supplementary Table 3. Quality assessment of miRNA studies according to QUADAS2 5](#_Toc531790321)

[Supplementary Table 4.The dysregulated miRNAs in single study 8](#_Toc531790317)

[Supplementary Table 5. The dysregulated miRNAs in tissue source 1](#_Toc531790317)0

[Supplementary Table 6. The dysregulated miRNAs in species subgroup 1](#_Toc531790317)0

[Supplementary Table 7. The dysregulated miRNAs in region subgroup 1](#_Toc531790317)0

**Figures**

[Supplementary Figure 1. Forest plot of miR-21 1](#_Toc531790322)1

[Supplementary Figure 2. Forest plot of miR-26a 1](#_Toc531790323)2

[Supplementary Figure 3.Forest plot of miR-29a 1](#_Toc531790324)3

[Supplementary Figure 4. Forest plot of miR-29c 1](#_Toc531790325)4

[Supplementary Figure 5. Forest plot of miR-34a 1](#_Toc531790326)5

[Supplementary Figure 6. Forest plot of miR-150 1](#_Toc531790327)6

[Supplementary Figure 7. Forest plot of miR-195 1](#_Toc531790328)7

[Supplementary Figure 8. Forest plot of miR-199a 1](#_Toc531790329)8

[Supplementary Figure 9. Forest plot of miR-200 1](#_Toc531790330)9

[Supplementary Figure 10. Forest plot of miR-208](#_Toc531790331) 20

[Supplementary Figure 11. Forest plot of miR-494 21](#_Toc531790332)

[Supplementary Figure 12. Forest plot of miR-141 22](#_Toc531790333)

[Supplementary Figure 13.Forest plot of let-7 23](#_Toc531790334)

[Supplementary Figure 14. Forest plot of miR-1 24](#_Toc531790335)

[Supplementary Figure 15. Forest plot of miR-26 25](#_Toc531790336)

[Supplementary Figure 16. Forest plot of miR-125b 26](#_Toc531790337)

[Supplementary Figure 17. Forest plot of miR-133 27](#_Toc531790338)

[Supplementary Figure 18. Forest plot of miR-143 2](#_Toc531790339)8

[Supplementary Figure 19. Forest plot of miR-378 2](#_Toc531790340)9

[Supplementary Figure 20. Forest plot of miR-503 30](#_Toc531790341)

**[References](#_Toc531790359)** [3](#_Toc531790359)1

# Supplementary Table 1. Search strategy used on October 28th, 2024

| **Literature databases** | **Search items** | **Items found** |
| --- | --- | --- |
| MEDLINE | (“microRNA”[MeSH Terms] OR “microRNA”[Title/Abstract] OR “miRNA”[Title/Abstract] OR “miR-”[Title/Abstract] )  AND [ (“glycometabolism”[Title/Abstract] OR “glycometabolism”[MeSH Terms] OR (“glucose metabolism”[Title/Abstract] OR “glucose metabolism”[MeSH Terms]] AND (“expression”[Title/Abstract] OR “profile”  [Title/Abstract] OR “profiling”[Title/Abstract]) | 1659 |
| EMBASE | (‘microRNA’/exp OR ‘microRNA’:ti, ab,kw OR ‘miRNA’: ti,ab,kw OR ‘miR-’: ti,ab,kw ) AND (‘glycometabolism’/exp OR ‘glycometabolism’:ti, ab,kw OR ‘glucose metabolism’: ti,ab,kw OR ‘glucose metabolism’:ti, ab,kw) AND (‘expression’ : ti,ab,kw OR ‘profile’: ti,ab,kw OR ‘profiling’: ti,ab,kw) | 92 |
| COCHRANE | (MeSH descriptor: [microRNA] OR microRNA: ti,ab,kw OR miRNA: ti,ab,kw OR miR-: ti,ab,kw) AND (MeSH descriptor: [glucose metabolism] OR glucose metabolism: ti,ab,kw (MeSH descriptor: [glycometabolism] OR glycometabolism: ti,ab,kw) AND (expression: ti,ab,kw OR profile: ti,ab,kw OR profiling: ti,ab,kw) | 29 |
| Overall |  | 1780 |

# Supplementary Table 2. Excluded studies with reasons

| **Study** | **Reason for exclusion** |
| --- | --- |
| Zhen et al. 2018^[1](#_ENREF_1)^ | Not glucose metabolism in the heart |
| Zhang et al. 2019^[2](#_ENREF_2)^ | Not reported sample size |
| Xiao et al. 2018^[3](#_ENREF_3)^ | Not glucose metabolism in the heart |
| Tattikota et al. 2015^[4](#_ENREF_4)^ | Not glucose metabolism in the heart |
| Rane et al. 2009^[5](#_ENREF_5)^ | No reported sample size |
| Mononen et al. 2019^[6](#_ENREF_6)^ | No reported sample size |
| Mirra et al. 2018^[7](#_ENREF_7)^ | Review |
| Guo et al. 2017^[8](#_ENREF_8)^ | Not glucose metabolism in the heart |
| Lynn et al. 2009^[9](#_ENREF_9)^ | Review |
| Luo et al. 2019^[10](#_ENREF_10)^ | No expression profiling |
| Luo et al. 2017^[11](#_ENREF_11)^ | Not glucose metabolism in the heart |
| Lin et al. 2016^[12](#_ENREF_12)^ | Not glucose metabolism in the heart |
| Liang et al. 2013^[13](#_ENREF_13)^ | No expression profiling |
| Li et al. 2020^[14](#_ENREF_14)^ | Not glucose metabolism in the heart |
| Li et al. 2015^[15](#_ENREF_15)^ | No expression profiling |
| Li et al. 2010^[16](#_ENREF_16)^ | Not glucose metabolism in the heart |
| Latouche et al. 2016^[17](#_ENREF_17)^ | Not glucose metabolism in the heart |
| Langlet et al. 2018^[18](#_ENREF_18)^ | Not glucose metabolism in the heart |
| Kornfeld et al. 2013^[19](#_ENREF_19)^ | No expression profiling |
| Katayama et al. 2019^[20](#_ENREF_20)^ | Not glucose metabolism in the heart |
| Julie Massart et al. 2017^[21](#_ENREF_21)^ | Not glucose metabolism in the heart |
| Jordan et al. 2011^[22](#_ENREF_22)^ | No expression profiling |
| Jiang et al. 2013^[23](#_ENREF_23)^ | No expression profiling |
| Guo et al. 2019^[24](#_ENREF_24)^ | Not glucose metabolism in the heart |
| Esteves et al. 2018^[25](#_ENREF_25)^ | Not glucose metabolism in the heart |
| Dumortier et al. 2020^[26](#_ENREF_26)^ | No expression profiling |
| Chuang et al. 2015^[27](#_ENREF_27)^ | Not glucose metabolism in the heart |
| Chen et al. 2013^[28](#_ENREF_28)^ | Not glucose metabolism in the heart |
| Chen et al. 2019^[29](#_ENREF_29)^ | No reported sample size |

# Supplementary Table 3. Quality assessment of miRNA studies according to QUADAS2.

| **Studies** | **Was a consecutive or random sample of patients enrolled?** | **Did the studyavoidinappropriate**  **exclusions?** | **Is the reference**  **standard likely to**  **correctly classify**  **the targetcondition?** | **Were the referencestandard resultsinterpreted without**  **knowledge of theresults of the indextest?** | **Was there an**  **appropriate interval**  **between index tests**  **and reference**  **standard?** | **Was the blind method used in result of referance standard?** | **Did allpatients**  **receive thesame reference**  **standard?** | **Were all**  **patients**  **included in**  **the analysis?** |
| --- | --- | --- | --- | --- | --- | --- | --- | --- |
| Zuo et al. 2016 | Y | U | Y | Y | Y | Y | Y | Y |
| Zhu et al. 2017 | Y | U | Y | Y | Y | Y | Y | Y |
| Zhang et al. 2017 | N | U | Y | Y | Y | Y | Y | Y |
| Zhang et al. 2013 | Y | N | Y | Y | Y | Y | Y | Y |
| Zhang et al. 2015 | N | N | Y | Y | Y | Y | Y | Y |
| Zhang et al. 2018 | Y | Y | Y | Y | Y | Y | Y | Y |
| Yu et al. 2021 | N | N | Y | Y | Y | Y | Y | Y |
| Yang T et al. 2019 | N | U | Y | Y | Y | Y | Y | Y |
| Yang Y et al. 2019 | U | U | Y | Y | Y | Y | Y | Y |
| Yan et al. 2015 | U | N | Y | Y | Y | Y | Y | Y |
| Xu et al. 2021 | Y | N | Y | Y | Y | Y | Y | Y |
| Wu J et al. 2019 | N | N | Y | Y | Y | Y | Y | Y |
| Wu N et al. 2019 | U | Y | Y | Y | Y | Y | Y | Y |
| Wu et al. 2023 | U | N | Y | Y | Y | Y | Y | Y |
| Wei et al. 2014 | U | U | Y | Y | Y | Y | Y | Y |
| Wang et al. 2009 | N | U | Y | Y | Y | Y | Y | Y |
| Wang et al. 2023 | N | N | Y | Y | Y | Y | Y | Y |
| Trotta et al. 2018 | U | Y | Y | Y | Y | Y | Y | Y |
| Ruiz-Velasco et al. 2020 | U | U | Y | Y | Y | Y | Y | Y |
| Park et al. 2018 | N | U | Y | Y | Y | Y | Y | Y |
| Nagalingam et al. 2013 | N | N | Y | Y | Y | Y | Y | Y |
| Mallat et al. 2014 | N | Y | Y | Y | Y | Y | Y | Y |
| Lu et al. 2010 | N | Y | Y | Y | Y | Y | Y | Y |
| Lu et al. 2020 | U | N | Y | Y | Y | Y | Y | Y |
| Long et al. 2013 | U | N | Y | Y | Y | Y | Y | Y |
| Liu et al. 2019 | N | U | Y | Y | Y | Y | Y | Y |
| Liu et al. 2020 | Y | U | Y | Y | Y | Y | Y | Y |
| Li et al. 2017 | Y | U | Y | Y | Y | Y | Y | Y |
| Li et al. 2020 | N | Y | Y | Y | Y | Y | Y | Y |
| Li et al. 2016 | N | Y | Y | Y | Y | Y | Y | Y |
| Lei et al. 2020 | N | Y | Y | Y | Y | Y | Y | Y |
| Kim et al. 2013 | Y | U | Y | Y | Y | Y | Y | Y |
| Ju et al. 2020 | U | N | Y | Y | Y | Y | Y | Y |
| Horie et al. 2009 | N | Y | Y | Y | Y | Y | Y | Y |
| He et al. 2014 | N | Y | Y | Y | Y | Y | Y | Y |
| Guedes et al. 2016 | N | Y | Y | Y | Y | Y | Y | Y |
| Gong et al. 2019 | Y | N | Y | Y | Y | Y | Y | Y |
| Fan et al. 2020 | Y | U | Y | Y | Y | Y | Y | Y |
| Du et al. 2015 | N | U | Y | Y | Y | Y | Y | Y |
| Dong et al. 2019 | U | N | Y | Y | Y | Y | Y | Y |
| Dong et al. 2018 | U | Y | Y | Y | Y | Y | Y | Y |
| Diao et al. 2011 | N | Y | Y | Y | Y | Y | Y | Y |
| Das et al. 2012 | Y | U | Y | Y | Y | Y | Y | Y |
| Borden et al. 2019 | Y | N | Y | Y | Y | Y | Y | Y |
| Baseler et al. 2012 | N | Y | Y | Y | Y | Y | Y | Y |
| Bartman et al. 2017 | N | Y | Y | Y | Y | Y | Y | Y |
| Arnold et al. 2014 | N | Y | Y | Y | Y | Y | Y | Y |

# Y: Yes; N: No; U: Unclear.

# Supplementary Table 4. The dysregulated miRNAs in single study.

| miRNA | Upregulated expressionNo. of studies No. of samples | Downregulated expressionNo. of studies No. of samples | | |
| --- | --- | --- | --- | --- |
| miR-99b-3p | 1 10 | miR-761 | 1 | 8 |
| miR-7225-5p | 1 6 | miR-499-5p | 1 | 10 |
| miR-705 | 1 6 | miR-450a-3p | 1 | 120 |
| miR-700 | 1 6 | miR-382-3p | 1 | 120 |
| miR-696 | 1 6 | miR-373 | 1 | 6 |
| miR-664 | 1 6 | miR-335 | 1 | 12 |
| miR-6240 | 1 6 | miR-322 | 1 | 6 |
| miR-499-3p | 1 6 | miR-3126-5p | 1 | 120 |
| miR-370 | 1 6 | miR-221-3p | 1 | 6 |
| miR-363–5p | 1 6 | miR-220b | 1 | 6 |
| miR-3470b | 1 6 | miR-218 | 1 | 6 |
| miR-3470a | 1 6 | miR-20a | 1 | 6 |
| miR-333 | 1 6 | miR-200a-3p | 1 | 20 |
| miR-327 | 1 6 | miR-1a-3p | 1 | 10 |
| miR-320 | 1 6 | miR-185-5p | 1 | 6 |
| miR-29b | 1 8 | miR-181b | 1 | 6 |
| miR-295 | 1 8 |  |  |  |
| miR-294 | 1 6 |  |  |  |
| miR-291–5p | 1 6 |  |  |  |
| miR-27a-3p | 1 6 |  |  |  |
| miR-24 | 1 6 |  |  |  |
| miR-223 | 1 12 |  |  |  |
| miR-221 | 1 6 |  |  |  |
| miR-21a-3p | 1 10 |  |  |  |
| miR-210 | 1 10 |  |  |  |
| miR-195a-3p | 1 10 |  |  |  |
| miR-187 | 1 12 |  |  |  |
| miR-181c | 1 10 |  |  |  |
| miR-155-5p | 1 6 |  |  |  |
| miR-154 | 1 24 |  |  |  |
| miR-144-3p | 1 10 |  |  |  |
| miR-142-3p | 1 6 |  |  |  |
| miR-135 | 1 18 |  |  |  |
| miR-129 | 1 6 |  |  |  |

# Supplementary Table 5. The dysregulated miRNAs in tissue source.

| Subgroup | direction | miRNA | No. of studies | No. of samples | **logOR** | 95%CI |
| --- | --- | --- | --- | --- | --- | --- |
|  |  | miR-21 | 4 | 42 | 4.69 | (2.63, 6.75) |
| myocardial tissue | up-regulated | miR-195 | 2 | 22 | 4.74 | (2.63, 6.75) |
|  |  | miR-208 | 2 | 14 | 4.15 | (1.20, 7.09) |
| myocardial cell | up-regulated | miR-29c | 2 | 18 | 4.60 | (1.69, 7.51) |
|  | down-regulated | miR-133 | 2 | 30 | 5.51 | (2.65, 8.37) |

# Supplementary Table 6. The dysregulated miRNAs in species subgroup.

| Subgroup | direction | miRNA | No. of studies | No. of samples | **logOR** | 95%CI |
| --- | --- | --- | --- | --- | --- | --- |
|  |  | miR-21 | 4 | 42 | 4.69 | (2.63, 6.75) |
| mouse | up-regulated | miR-29a | 2 | 14 | 4.15 | (1.20, 7.09) |
|  |  | miR-208 | 2 | 14 | 4.15 | (1.20, 7.09) |

# Supplementary Table 7. The dysregulated miRNAs in region subgroup.

| Subgroup | direction | miRNA | No. of studies | No. of samples | **logOR** | 95%CI |
| --- | --- | --- | --- | --- | --- | --- |
|  |  | miR-199a | 7 | 82 | 4.59 | (3.02, 6.15) |
| Asian | up-regulated | miR-494 | 3 | 18 | 4.73 | (2.36, 7.10) |
|  |  | miR-26a | 3 | 76 | 6.35 | (4.04, 8.67) |
|  |  | miR-195 | 2 | 22 | 4.74 | (1.83, 7.65) |
|  |  | miR-26 | 2 | 32 | 5.67 | (2.81, 8.52) |
|  | down-regulated | miR-125b | 2 | 16 | 4.35 | (1.43, 7.82) |
|  |  | miR-133 | 2 | 30 | 5.51 | (2.65, 8.37) |
|  |  | miR-503 | 2 | 12 | 3.89 | (0.93, 6.86) |
|  | up-regulated | miR-29c | 2 | 18 | 4.60 | (1.69, 7.51) |
| Non-Asian |  | miR-141 | 2 | 16 | 4.60 | (1.69, 7.51) |
|  | down-regulated | miR-378 | 3 | 28 | 4.62 | (2.24, 7.00) |

**
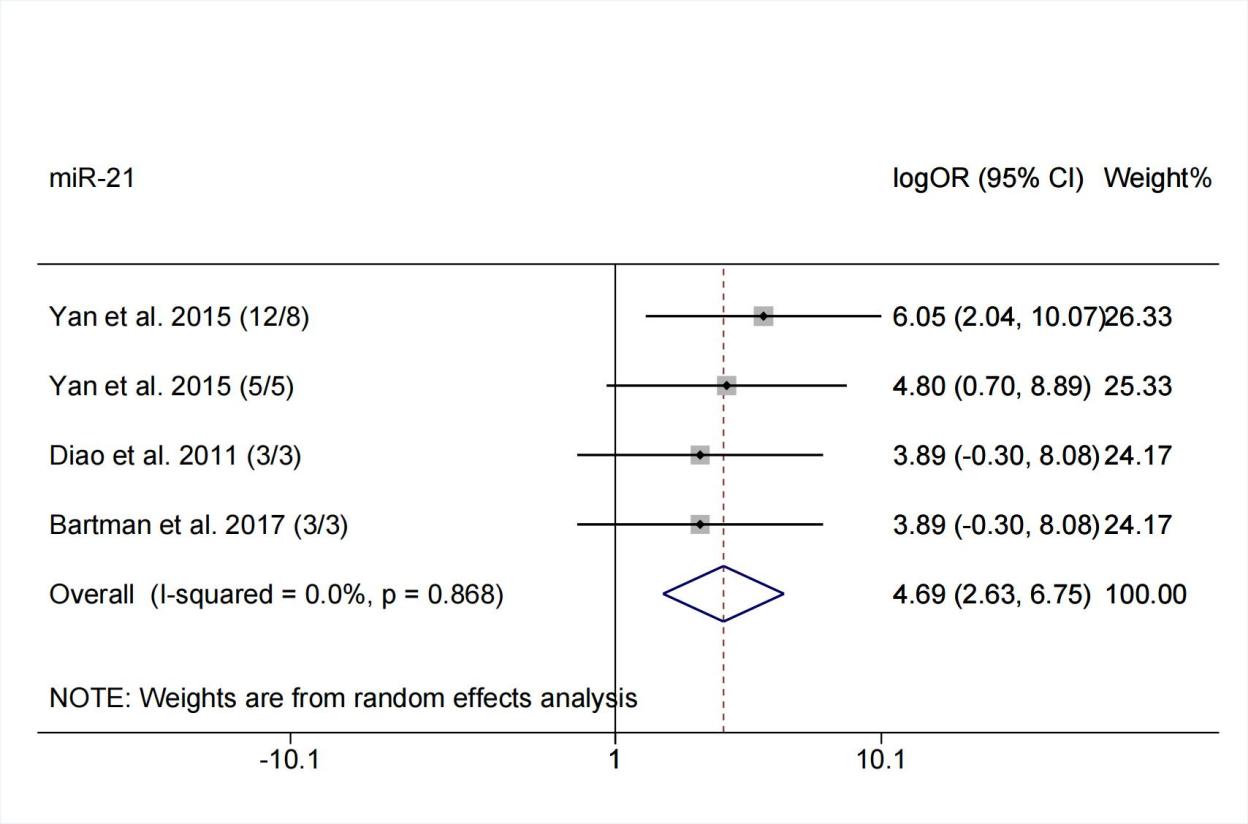
**

# Supplementary Figure1. Forest plot of miR-21

**
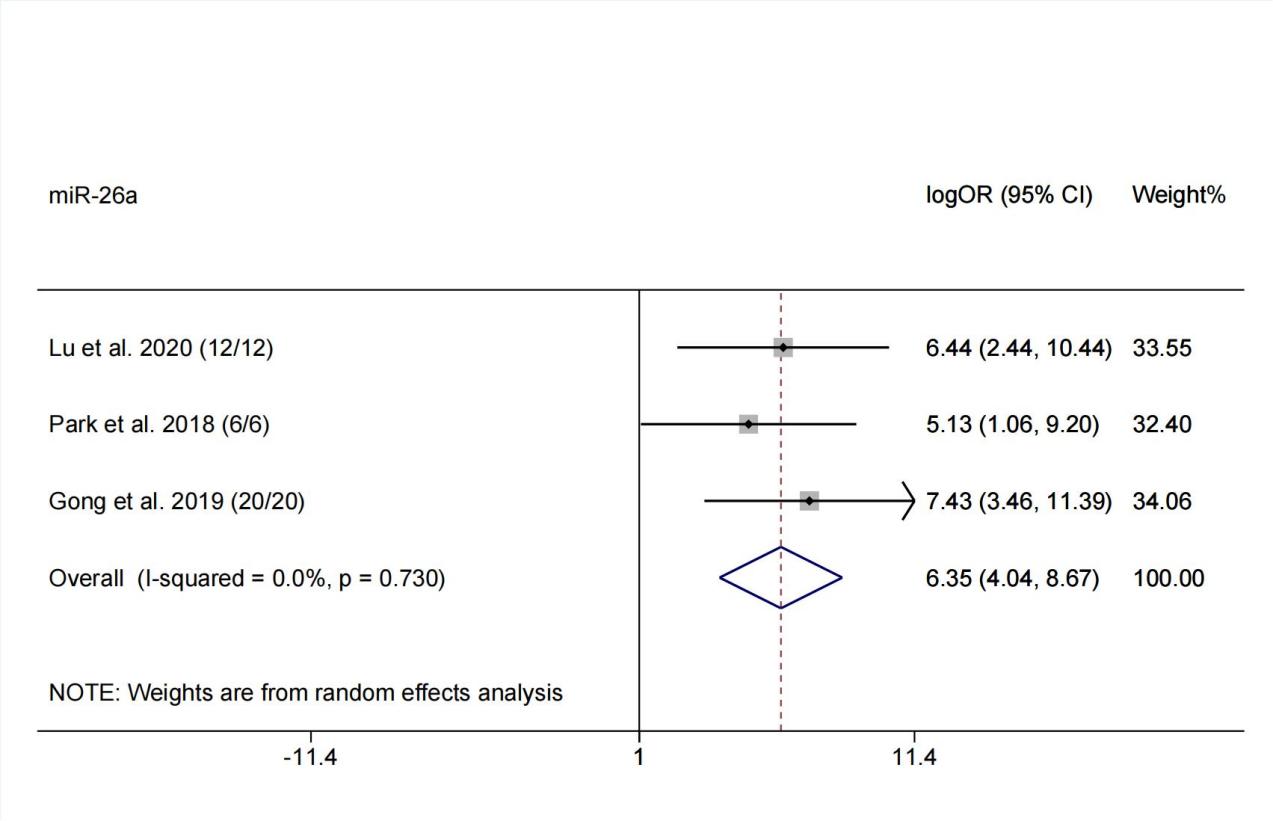
**

# Supplementary Figure2. Forest plot of miR-26

**
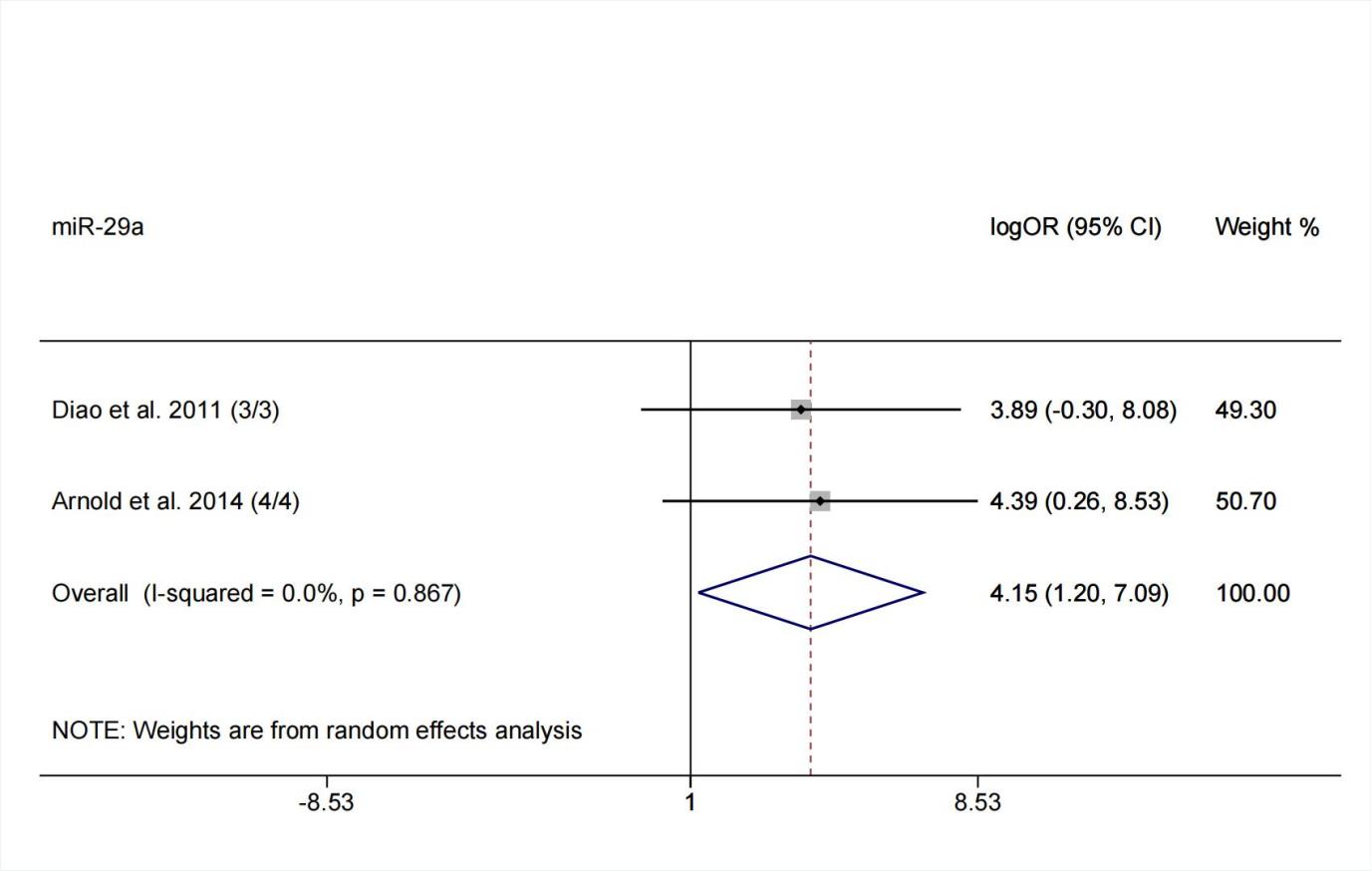
**

# Supplementary Figure3. Forest plot of miR-29a

**
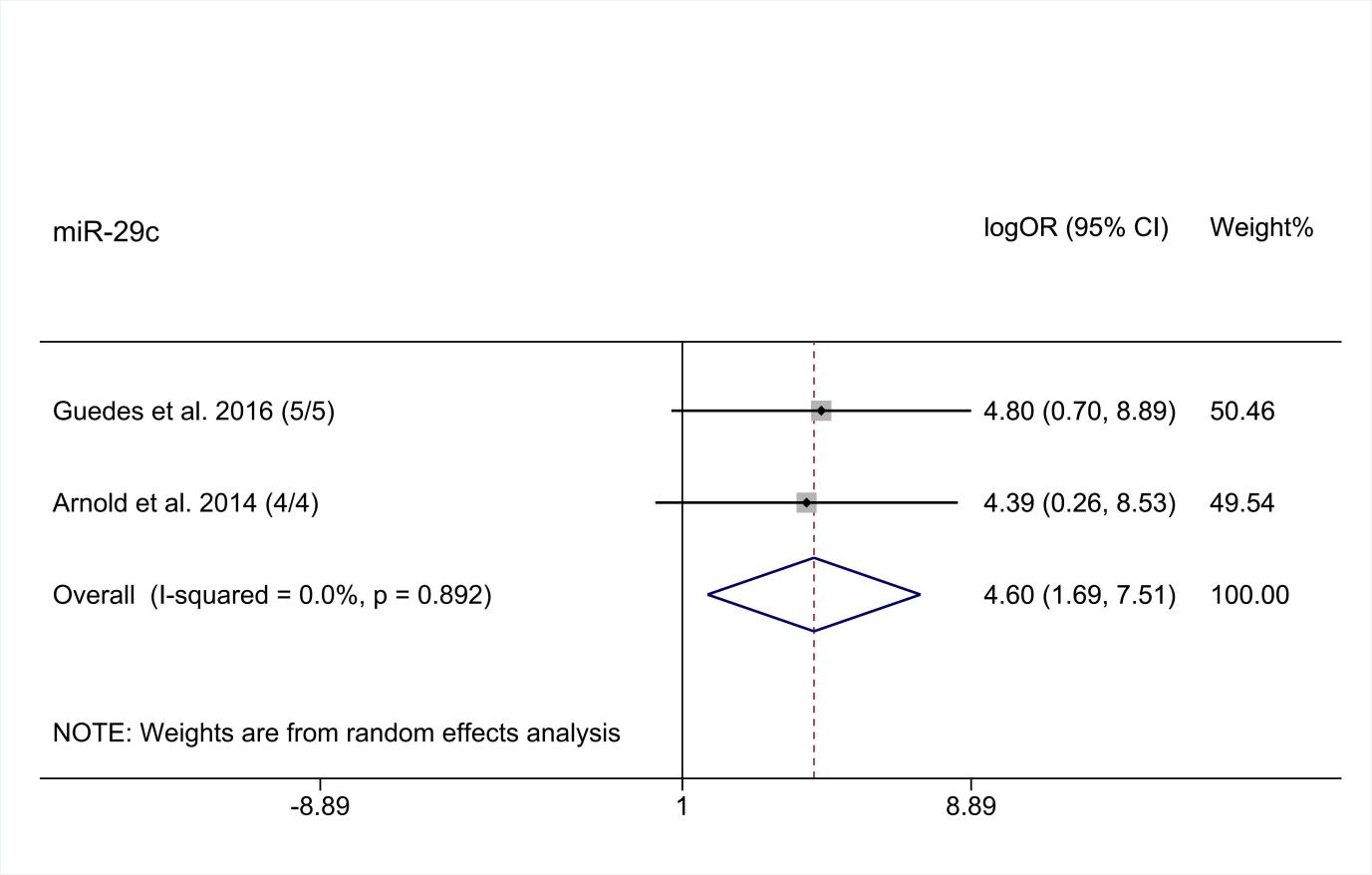
**

# Supplementary Figure4. Forest plot of miR-29c

**
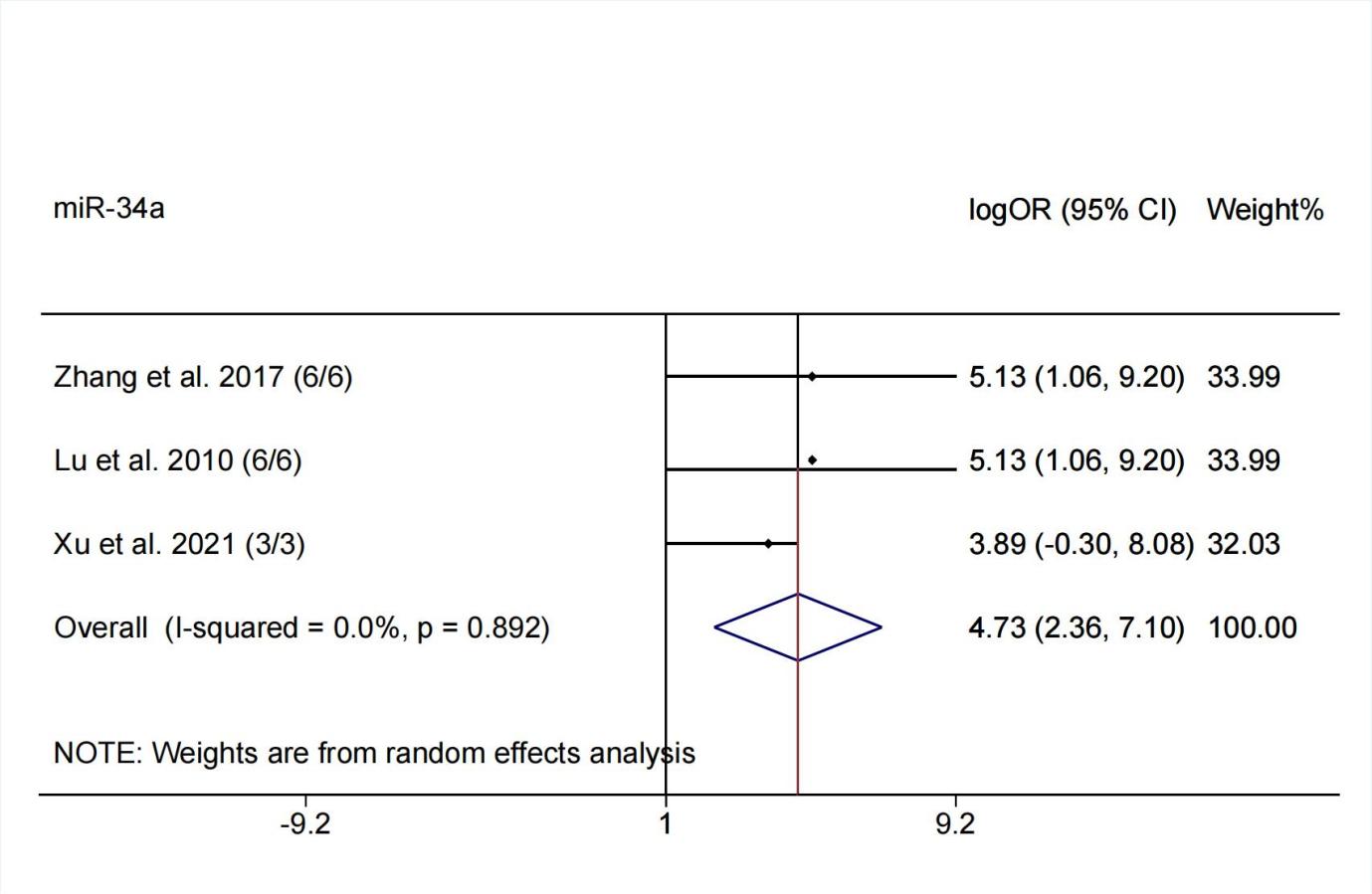
**

# Supplementary Figure5. Forest plot of miR-34a

**
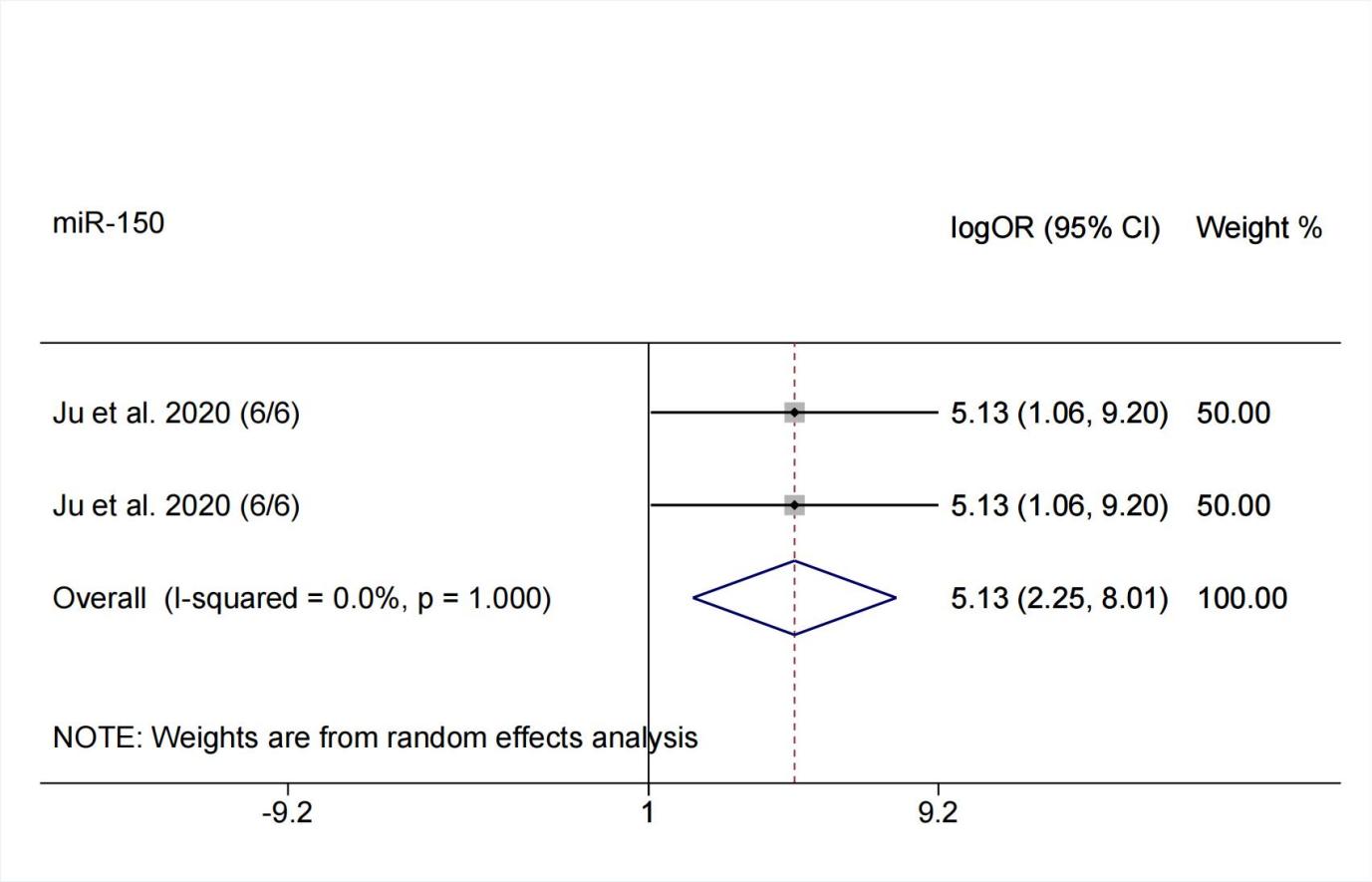
**

# Supplementary Figure6. Forest plot of miR-150

**
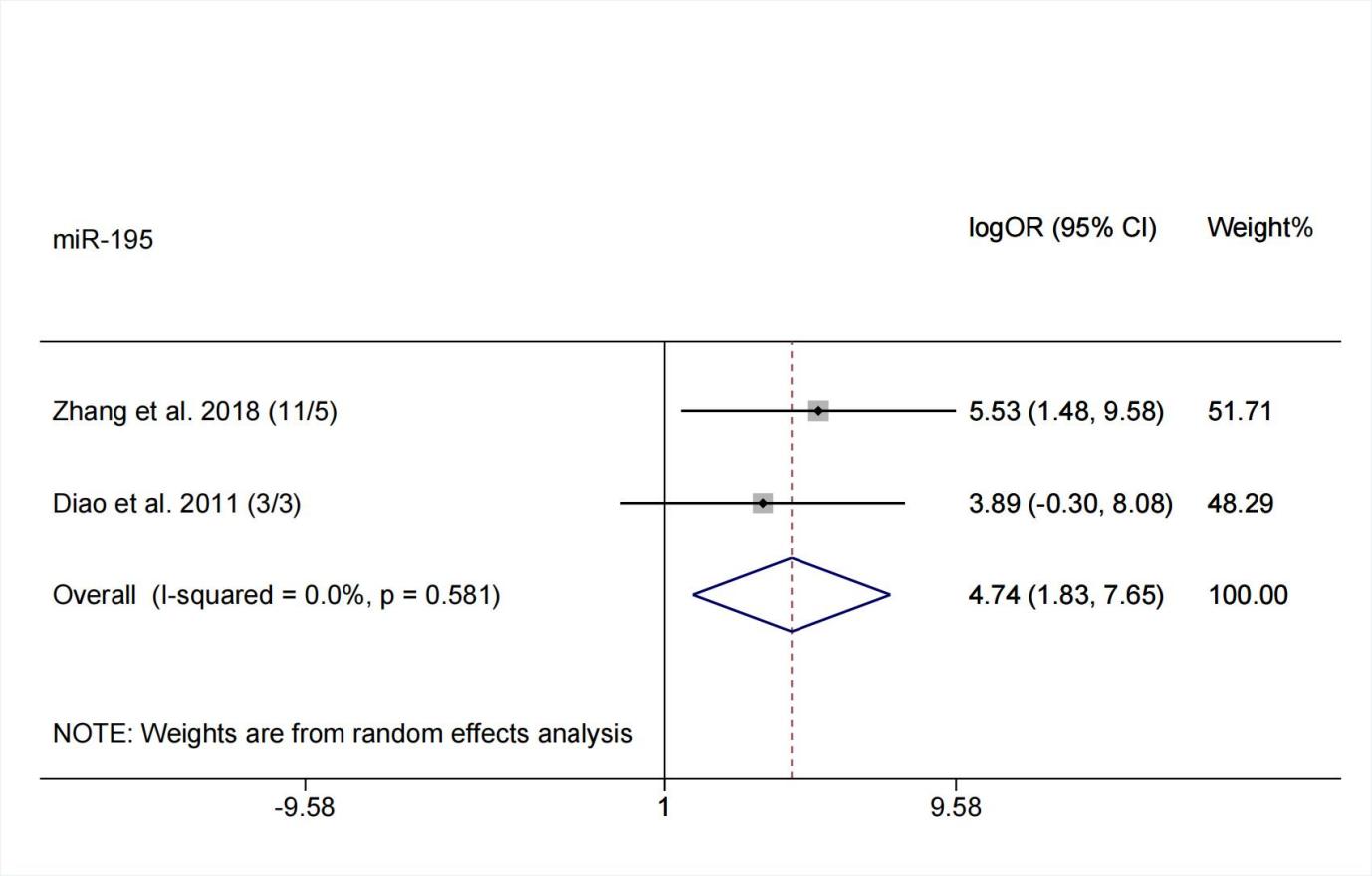
**

# Supplementary Figure7. Forest plot of miR-195

**
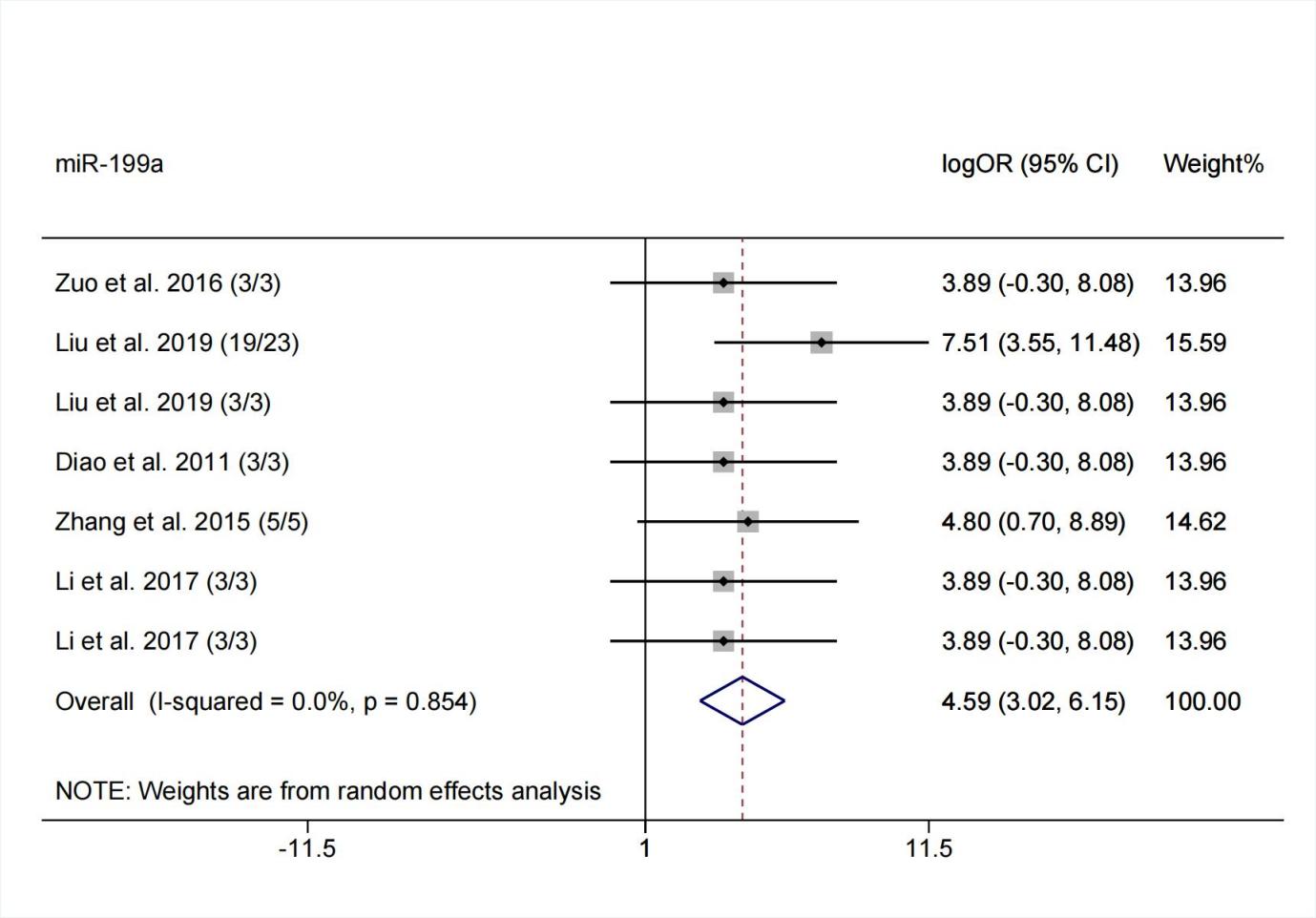
**

# Supplementary Figure8. Forest plot of miR-199a


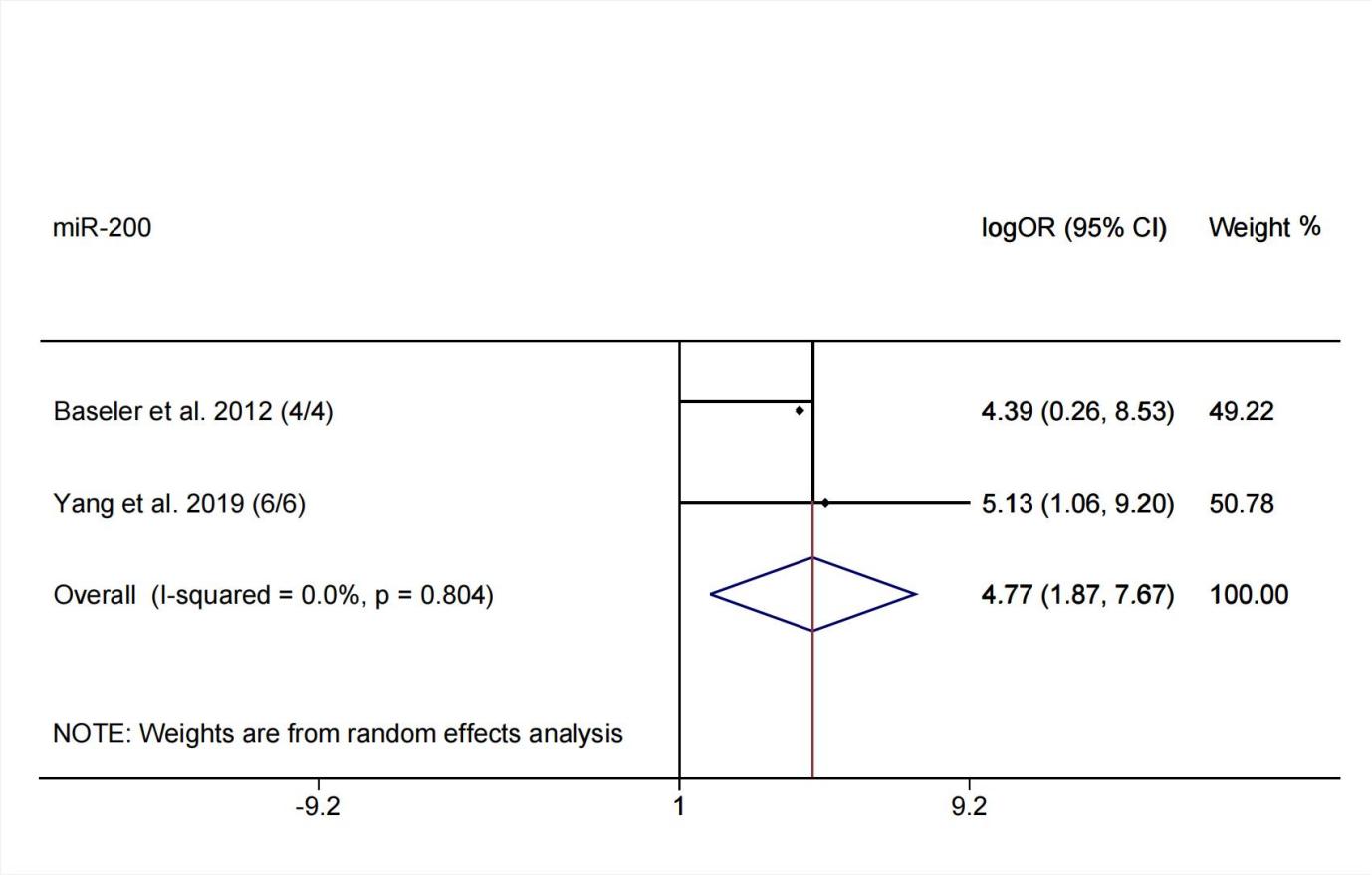


# Supplementary Figure9. Forest plot of miR-200

**
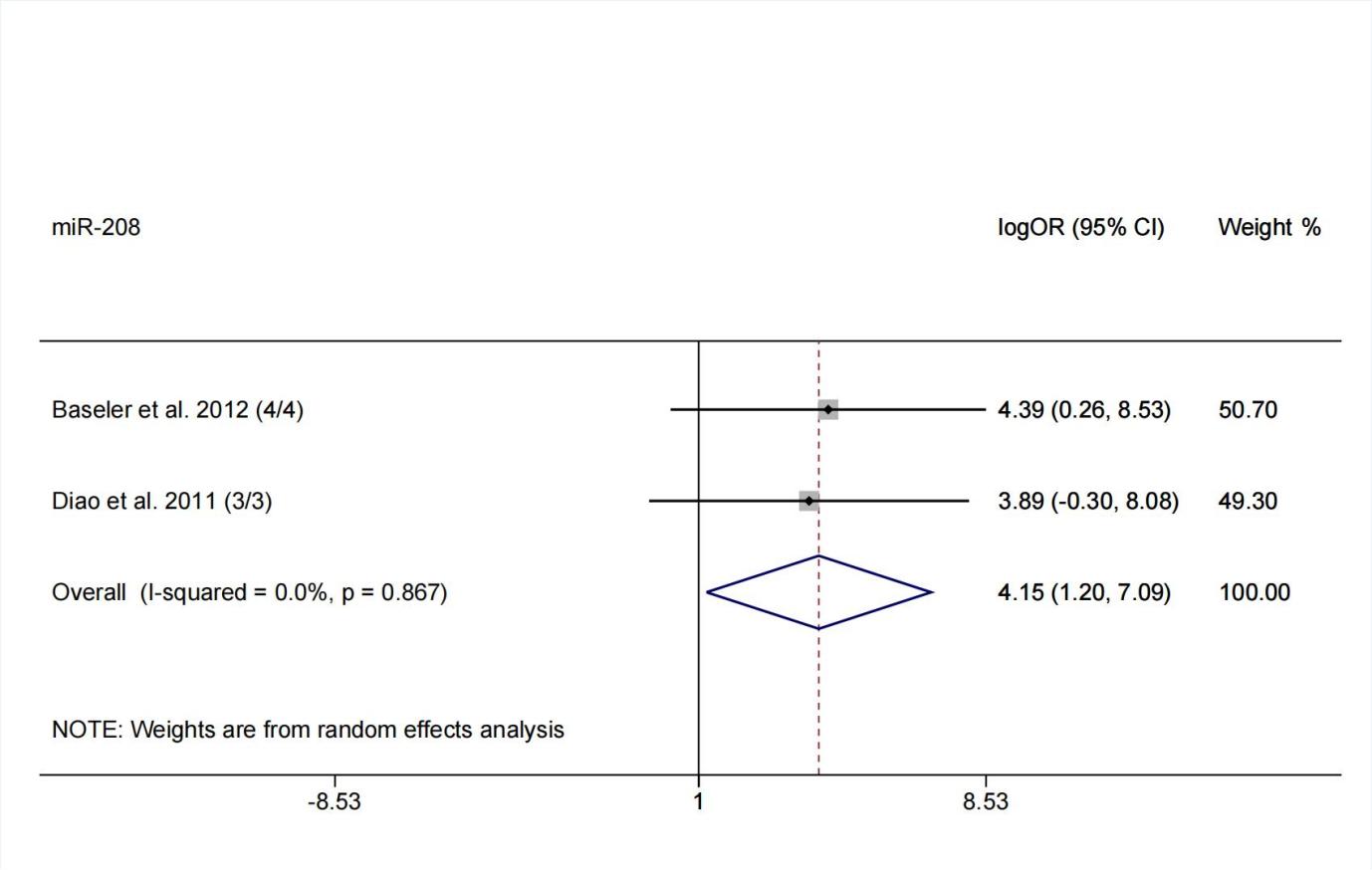
**

# Supplementary Figure10. Forest plot of miR-208

**
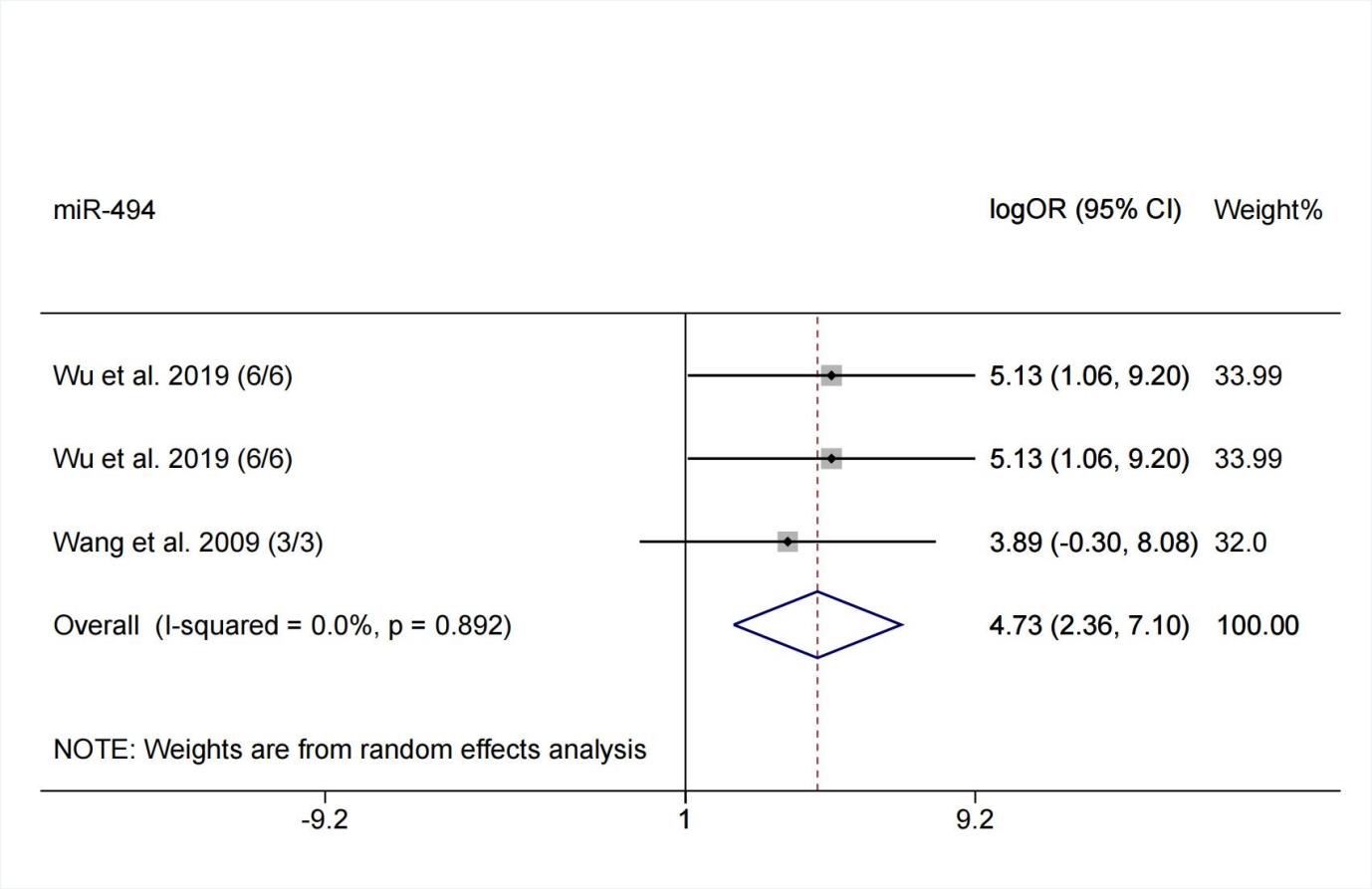
**

# Supplementary Figure11. Forest plot of miR-494

**
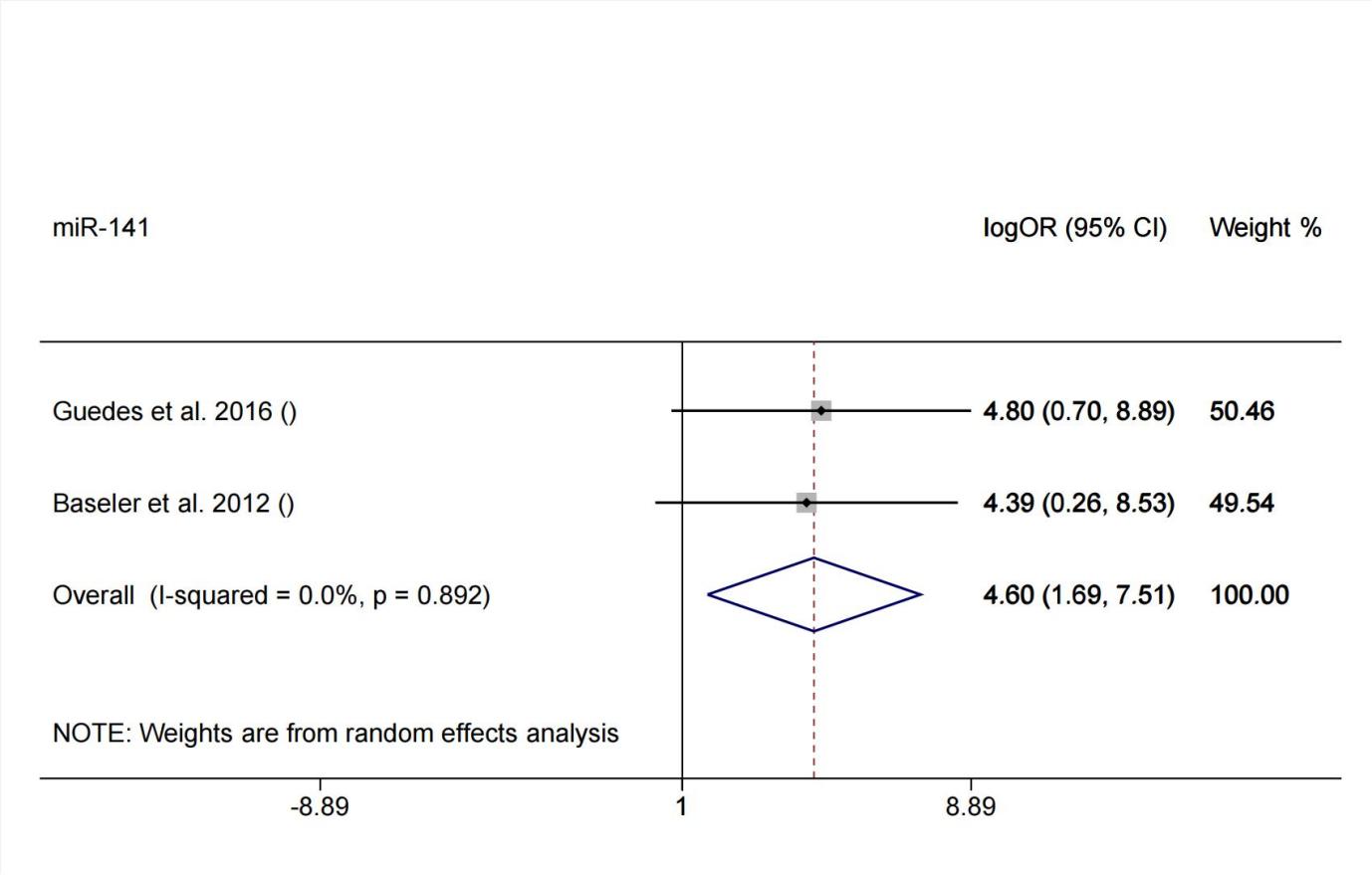
**

# Supplementary Figure12. Forest plot of miR-141

**
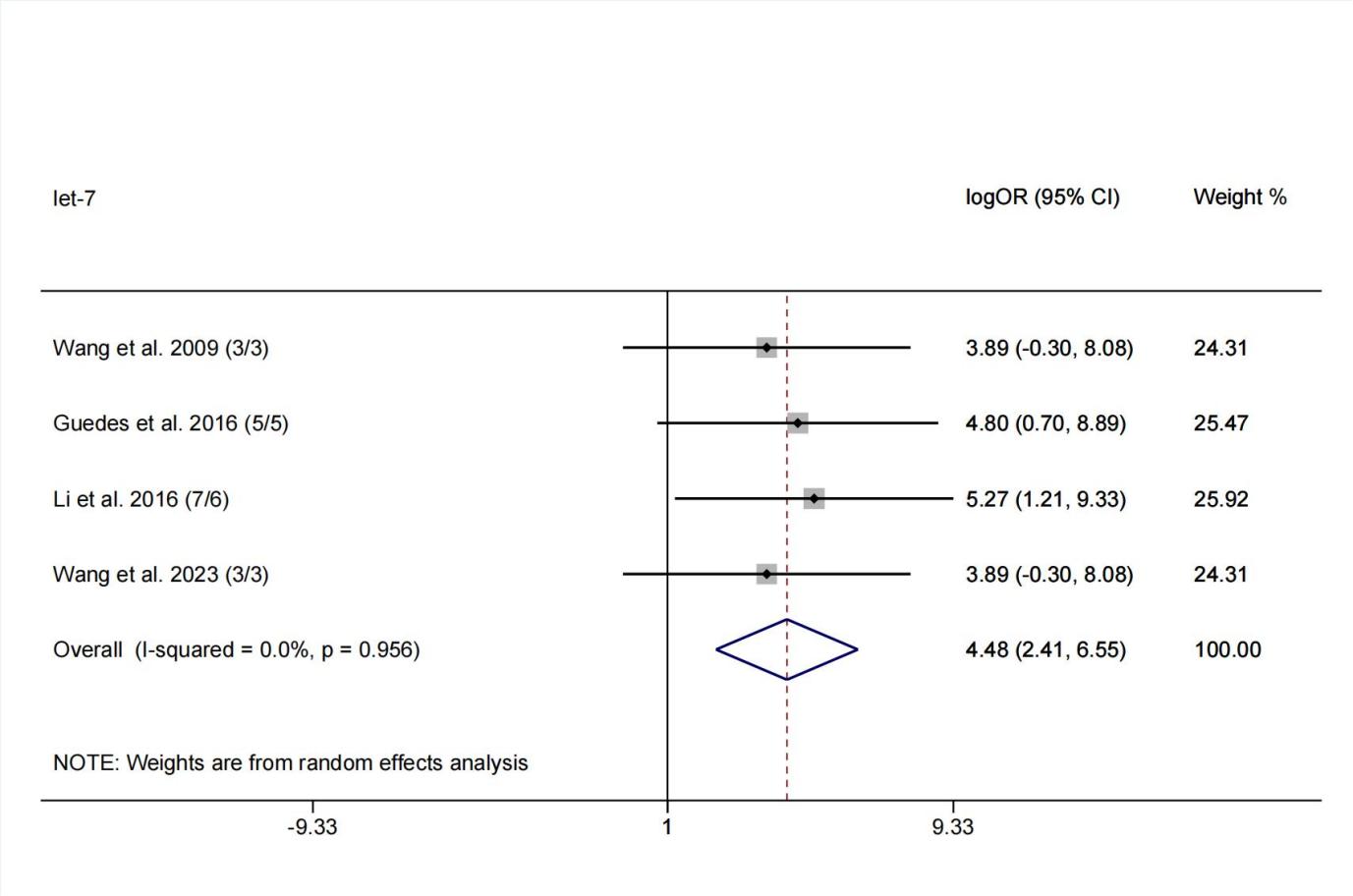
**

# Supplementary Figure13. Forest plot of let-7

**
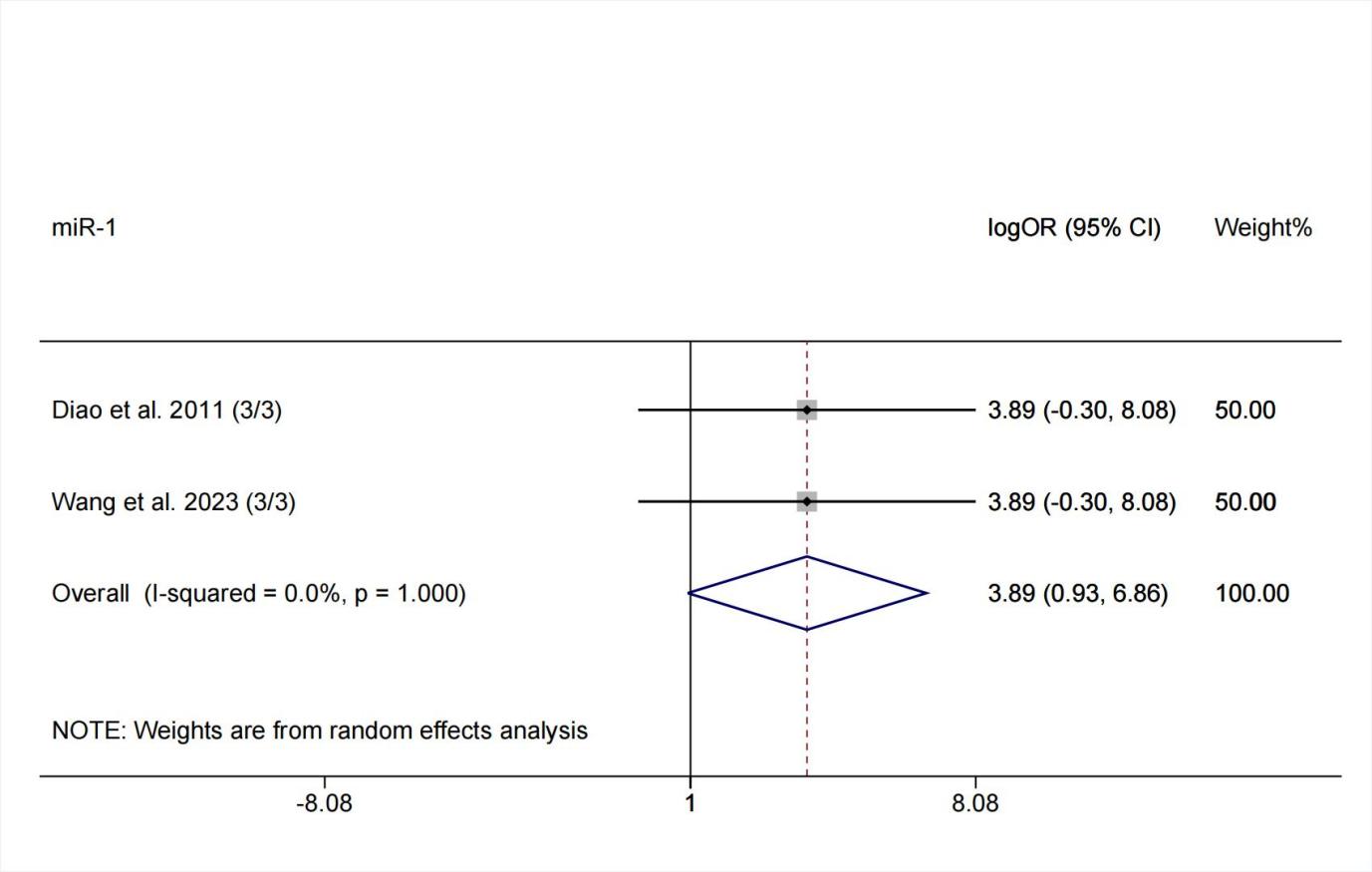
**

# Supplementary Figure14. Forest plot of miR-1

**
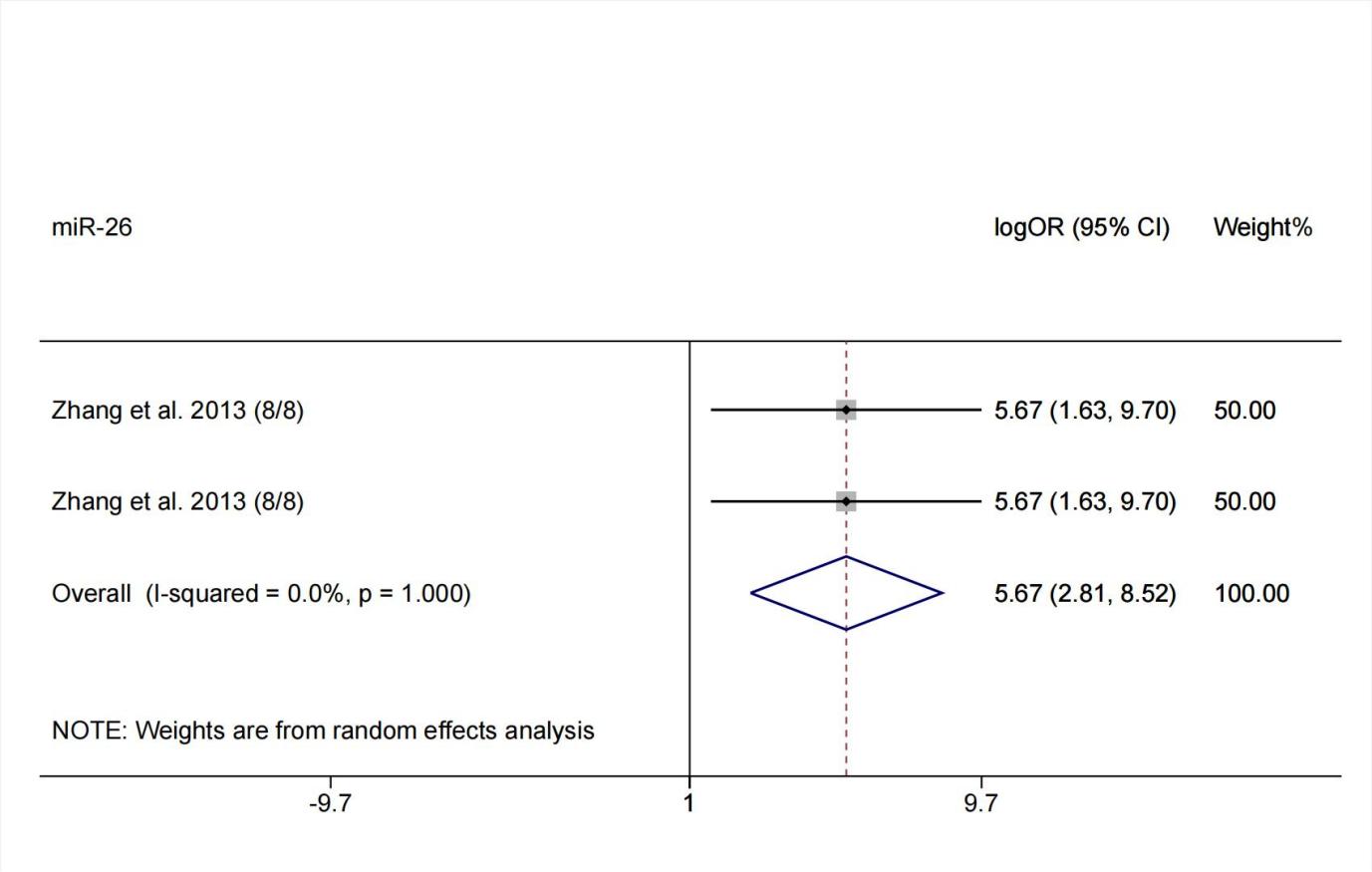
**

# Supplementary Figure15. Forest plot of miR-26

**
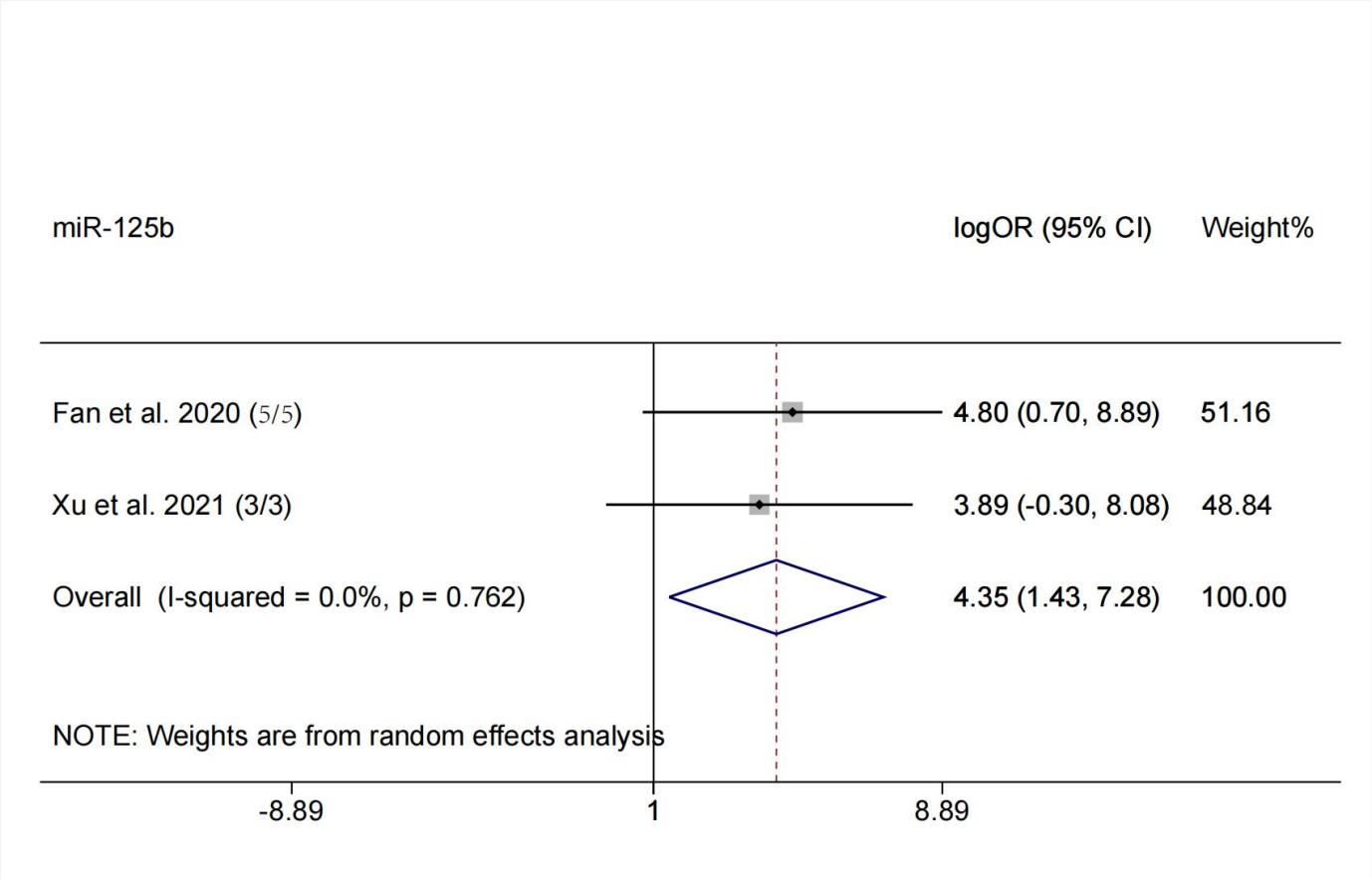
**

# Supplementary Figure16. Forest plot of miR-125b

**
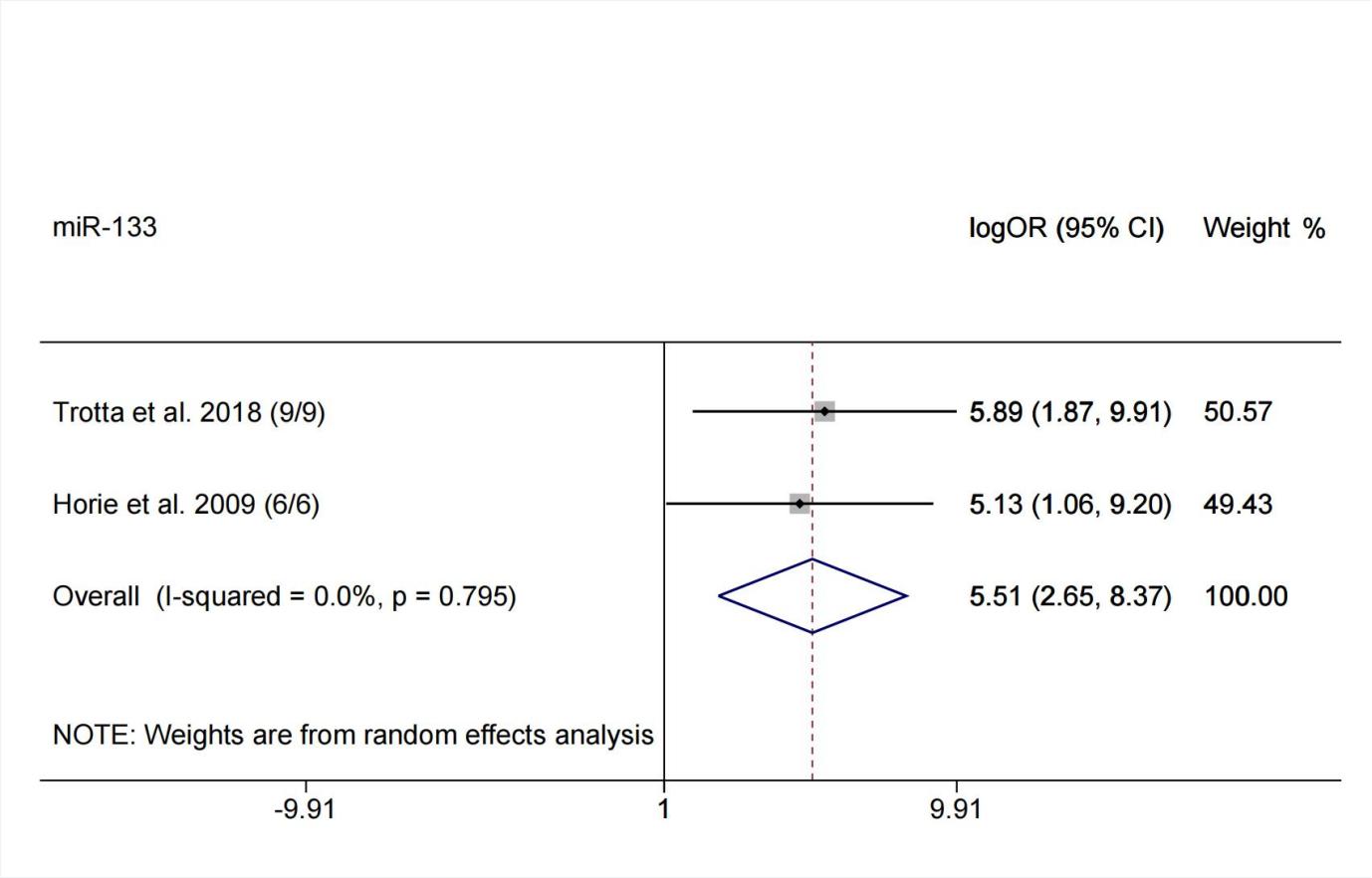
**

# Supplementary Figure17. Forest plot of miR-133

**
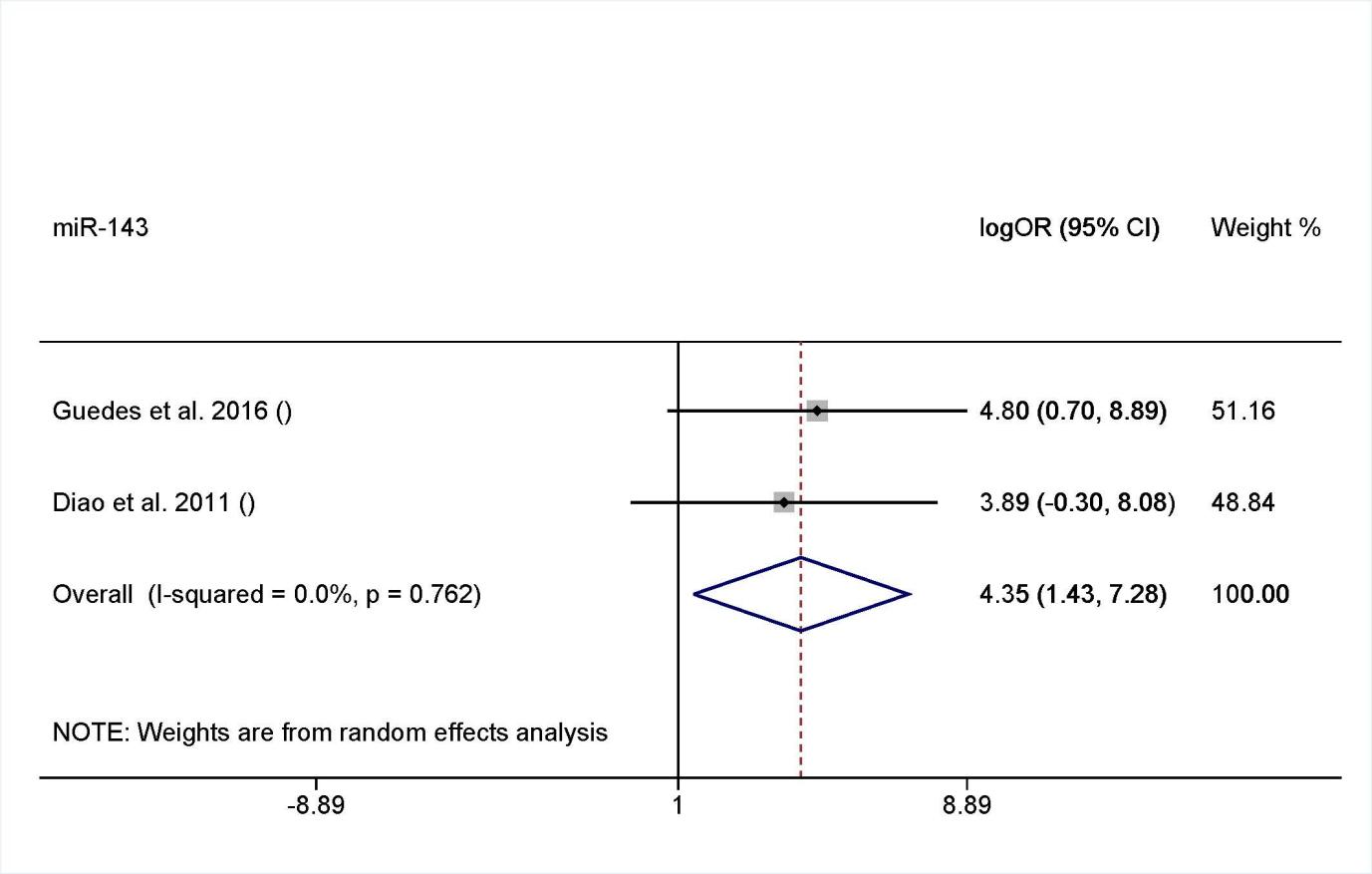
**

# Supplementary Figure 18. Forest plot of miR-143

#

#
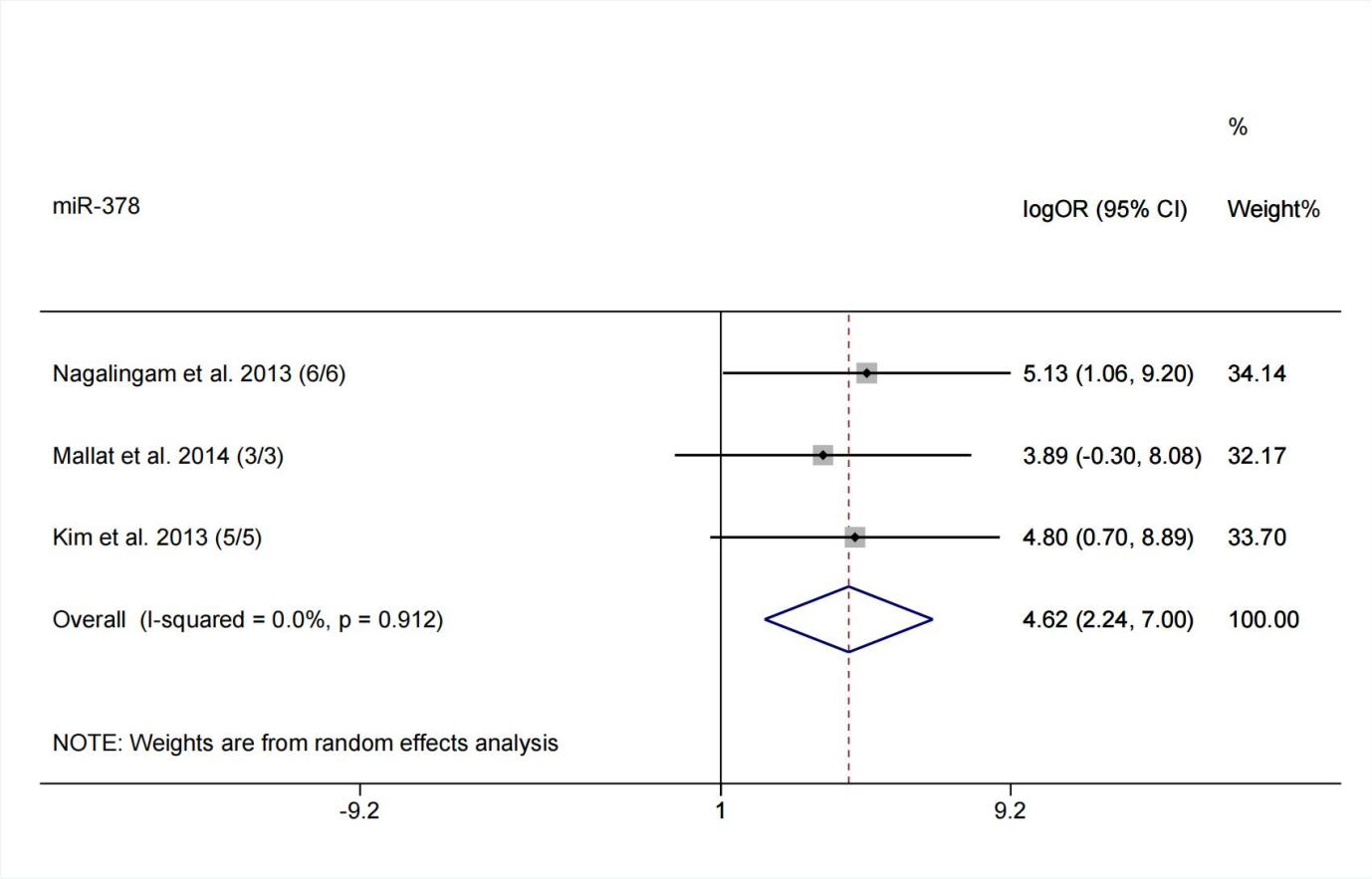


# Supplementary Figure19. Forest plot of miR-378

#
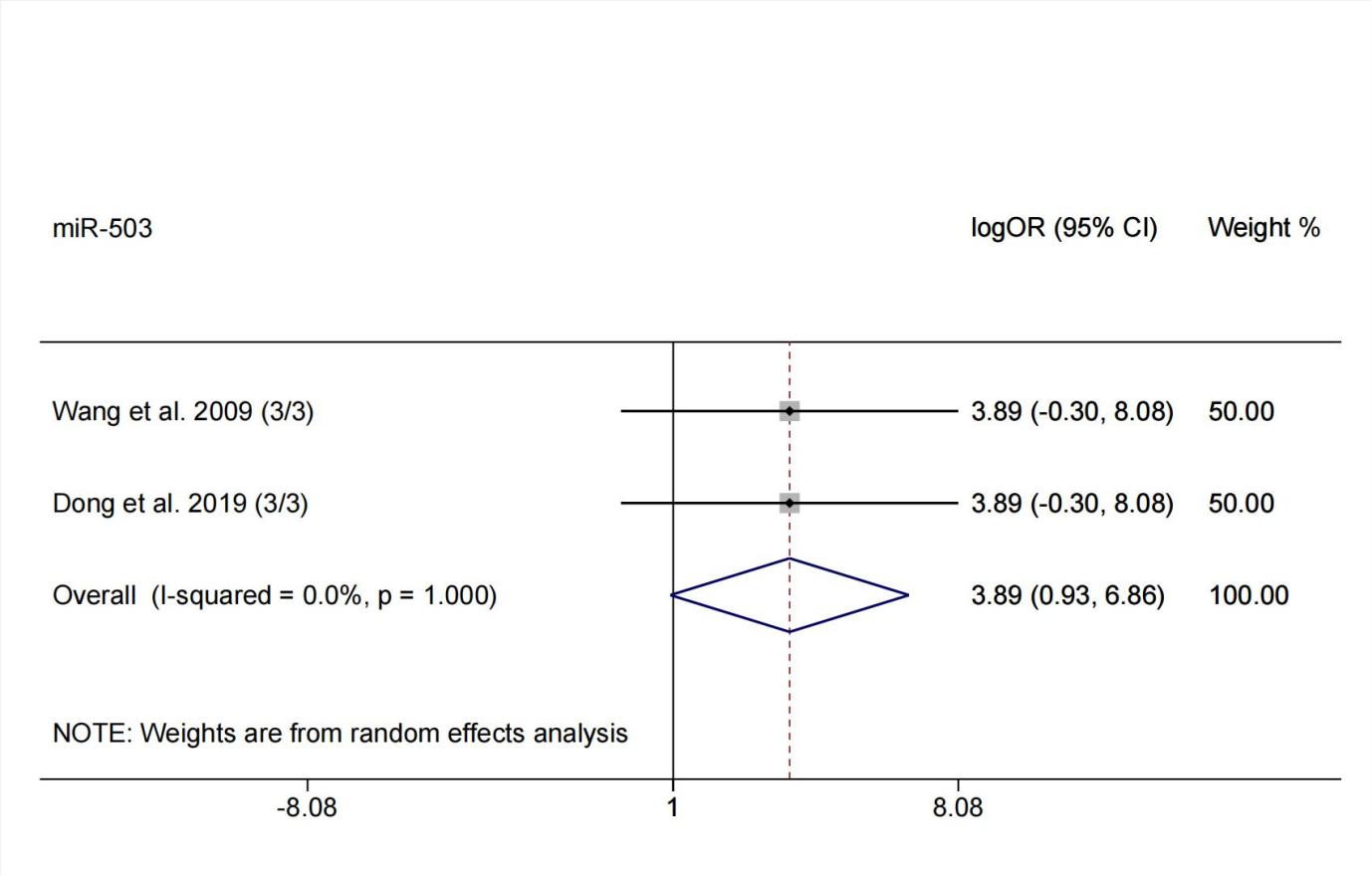


# Supplementary Figure20. Forest plot of miR-503

# References

1. Zhen YF, Zhang YJ, Zhao H, Ma HJ, Song GY. Microrna-802 regulates hepatic insulin sensitivity and glucose metabolism. *International journal of clinical and experimental pathology*. 2018;11:2440-2449

2. Zhang BH, Shen CA, Zhu BW, An HY, Zheng B, Xu SB, Sun JC, Sun PC, Zhang W, Wang J, Liu JY, Fan YQ. Insight into mirnas related with glucometabolic disorder. *Biomedicine & pharmacotherapy = Biomedecine & pharmacotherapie*. 2019;111:657-665

3. Xiao D, Zhou T, Fu Y, Wang R, Zhang H, Li M, Lin Y, Li Z, Xu C, Yang B, Zhang Y, Zhang Y. Microrna-17 impairs glucose metabolism in insulin-resistant skeletal muscle via repressing glucose transporter 4 expression. *European journal of pharmacology*. 2018;838:170-176

4. Tattikota SG, Rathjen T, Hausser J, Khedkar A, Kabra UD, Pandey V, Sury M, Wessels HH, Mollet IG, Eliasson L, Selbach M, Zinzen RP, Zavolan M, Kadener S, Tschop MH, Jastroch M, Friedlander MR, Poy MN. Mir-184 regulates pancreatic beta-cell function according to glucose metabolism. *The Journal of biological chemistry*. 2015;290:20284-20294

5. Rane S, He M, Sayed D, Vashistha H, Malhotra A, Sadoshima J, Vatner DE, Vatner SF, Abdellatif M. Downregulation of mir-199a derepresses hypoxia-inducible factor-1alpha and sirtuin 1 and recapitulates hypoxia preconditioning in cardiac myocytes. *Circ Res*. 2009;104:879-886

6. Mononen N, Lyytikainen LP. Whole blood microrna levels associate with glycemic status and correlate with target mrnas in pathways important to type 2 diabetes. 2019;9:8887

7. Mirra P, Nigro C, Prevenzano I, Leone A, Raciti GA, Formisano P, Beguinot F, Miele C. The destiny of glucose from a microrna perspective. *Journal of cellular biochemistry*. 2018;9:46

8. Mauer J, Baitzel C, Hansmeier N, Khani S, Konieczka S, Pradas-Juni M, Brodesser S, Van TM, Bartsch D, Bronneke HS, Heine M, Hilpert H, Tarcitano E, Garinis GA, Frommolt P, Heeren J, Mori MA, Bruning JC, Kornfeld JW, Guo J, Dou L, Meng X, Chen Z, Yang W, Fang W, Yang C, Huang X, Tang W, Yang J, Li J. Hepatic mir-291b-3p mediated glucose metabolism by directly targeting p65 to upregulate pten expression. *Nature cell biology*. 2017;7:39899

9. Lynn FC. Meta-regulation: Microrna regulation of glucose and lipid metabolism. *Trends in endocrinology and metabolism: TEM*. 2009;20:452-459

10. Luo M, Xu C, Luo Y, Wang G, Wu J, Wan Q. Circulating mir-103 family as potential biomarkers for type 2 diabetes through targeting cav-1 and sfrp4. 2019

11. Luo A, Yan H, Liang J, Du C, Zhao X, Sun L, Chen Y. Microrna-21 regulates hepatic glucose metabolism by targeting foxo1. *Journal of cellular physiology*. 2017;627:194-201

12. Lin X, Qin Y, Jia J, Lin T, Lin X, Chen L, Zeng H, Han Y, Wu L, Huang S, Wang M, Huang S, Xie R, Liang L, Liu Y, Liu R, Zhang T, Li J, Wang S, Sun P, Huang W, Yao K, Xu K, Du T, Xiao D. Mir-155 enhances insulin sensitivity by coordinated regulation of multiple genes in mice. *PLoS genetics*. 2016;12:e1006308

13. Liang J, Liu C, Qiao A, Cui Y, Zhang H, Cui A, Zhang S, Yang Y, Xiao X, Chen Y, Fang F, Chang Y. Microrna-29a-c decrease fasting blood glucose levels by negatively regulating hepatic gluconeogenesis. *Journal of hepatology*. 2013;58:535-542

14. Li K, Zhao B, Wei D, Wang W, Cui Y, Qian L, Liu G. Mir146a improves hepatic lipid and glucose metabolism by targeting med1. *International journal of molecular medicine*. 2020;45:543-555

15. Li K, Zhang J, Yu J, Liu B, Guo Y, Deng J, Chen S, Wang C, Guo F. Microrna-214 suppresses gluconeogenesis by targeting activating transcriptional factor 4. *The Journal of biological chemistry*. 2015;290:8185-8195

16. Li J, Donath S, Li Y, Qin D, Prabhakar BS, Li P. Mir-30 regulates mitochondrial fission through targeting p53 and the dynamin-related protein-1 pathway. *PLoS genetics*. 2010;6:e1000795

17. Latouche C, Natoli A, Reddy-Luthmoodoo M, Heywood SE, Armitage JA, Kingwell BA. Microrna-194 modulates glucose metabolism and its skeletal muscle expression is reduced in diabetes. *Journal of cellular physiology*. 2016;11:e0155108

18. Langlet F, Tarbier M, Haeusler RA, Camastra S, Ferrannini E, Friedlander MR, Accili D. Microrna-205-5p is a modulator of insulin sensitivity that inhibits foxo function. *Molecular metabolism*. 2018;17:49-60

19. Kornfeld JW, Baitzel C, Konner AC, Nicholls HT, Vogt MC, Herrmanns K, Scheja L, Haumaitre C, Wolf AM, Knippschild U, Seibler J, Cereghini S, Heeren J, Stoffel M, Bruning JC. Obesity-induced overexpression of mir-802 impairs glucose metabolism through silencing of hnf1b. *Nature*. 2013;494:111-115

20. Katayama M, Wiklander OPB, Fritz T, Caidahl K, El-Andaloussi S, Zierath JR. Circulating exosomal mir-20b-5p is elevated in type 2 diabetes and could impair insulin action in human skeletal muscle. 2019;68:515-526

21. Julie Massar, Rasmus J.O. Sjögren, Lundell LS, Mudry JM, Franck N. Altered mir-29 expression in type 2 diabetes influences glucose and lipid metabolism in skeletal muscle. *Oncogene*. 2017;66:1807-1818

22. Jordan SD, Kruger M, Willmes DM, Redemann N, Wunderlich FT, Bronneke HS, Merkwirth C, Kashkar H, Olkkonen VM, Bottger T, Braun T, Seibler J, Bruning JC. Obesity-induced overexpression of mirna-143 inhibits insulin-stimulated akt activation and impairs glucose metabolism. *Nature cell biology*. 2011;13:434-446

23. Jiang LQ, Franck N, Egan B, Sjogren RJ, Katayama M, Duque-Guimaraes D, Arner P, Zierath JR, Krook A. Autocrine role of interleukin-13 on skeletal muscle glucose metabolism in type 2 diabetic patients involves microrna let-7. *American journal of physiology. Endocrinology and metabolism*. 2013;305:E1359-1366

24. Guo Y, Li G, Li H, Huang C, Liu Q, Dou Y, Yin X, Dong L, Yang N, Han Z. Microrna-15a inhibits glucose transporter 4 translocation and impairs glucose metabolism in l6 skeletal muscle via targeting of vesicle-associated membrane protein-associated protein a. *Canadian journal of diabetes*. 2019

25. Esteves JV, Yonamine CY, Pinto-Junior DC, Gerlinger-Romero F, Enguita FJ, Machado UF. Diabetes modulates micrornas 29b-3p, 29c-3p, 199a-5p and 532-3p expression in muscle: Possible role in glut4 and hk2 repression. *Frontiers in endocrinology*. 2018;9:536

26. Dumortier O, Fabris G, Pisani DF, Casamento V, Gautier N, Hinault C, Lebrun P, Duranton C, Tauc M, Dalle S, Kerr-Conte J, Pattou F, Prentki M, Obberghen EV. Microrna-375 regulates glucose metabolism-related signaling for insulin secretion. *Diabetes*. 2020;244:189-200

27. Chuang TY, Wu HL, Chen CC, Gamboa GM, Layman LC, Diamond MP, Azziz R, Chen YH. Microrna-223 expression is upregulated in insulin resistant human adipose tissue. *Journal of diabetes research*. 2015;2015:943659

28. Chen YH, Heneidi S, Lee JM, Layman LC, Stepp DW, Gamboa GM, Chen BS, Chazenbalk G, Azziz R. Mirna-93 inhibits glut4 and is overexpressed in adipose tissue of polycystic ovary syndrome patients and women with insulin resistance. *Diabetes*. 2013;62:2278-2286

29. Chen T, Zhang Y, Liu Y, Zhu D, Yu J, Li G, Sun Z, Wang W, Jiang H, Hong Z. Mir-27a promotes insulin resistance and mediates glucose metabolism by targeting ppar-gamma-mediated pi3k/akt signaling. *Aging*. 2019;11:7510-7524
